# Supplementary material for: Tenascin C Promotes Hematoendothelial Development and T Lymphoid Commitment from Human Pluripotent Stem Cells in Chemically Defined Conditions
Source: Stem Cell Reports. 2014 Oct 23;3(6):1073–84. doi: 10.1016/j.stemcr.2014.09.014 (PMC4263995; doi:10.1016/j.stemcr.2014.09.014)
Supplement: Document S1. Supplemental Experimental Procedures, Figures S1–S5, and Table S1 [file mmc1.pdf]

Stem Cell Reports, Volume *3*

Supplemental Information

Tenascin C Promotes Hematoendothelial Development and T Lymphoid Commitment from Human Pluripotent Stem Cells in Chemically Defined Conditions

Gene Uenishi, Derek Theisen, Jeong-Hee Lee, Akhilesh Kumar, Matt Raymond, Maxim Vodyanik, Scott Swanson, Ron Stewart, James Thomson, and Igor Slukvin

Supplementary Experimental Procedures

Flow Cytometry and FACS sorting

Flow Cytometry was conducted using the using a FACSCalibur flow cytometer (BD) and following antibodies: CD31-FITC (clone WM59), CD34-FITC (8G12), CD41a-FITC/APC (clone HIP8), CD43-FITC/PE/APC (clone 1G10), CD45-APC (clone HI30), CD73-FITC/PE (clone AD2), CD144-FITC/PE/AlexaFluor647 (clone 55-7H1), CD235a-FITC/PE/APC (clone GA-R2), KDR-PE/AlexaFluor647 (clone 89106), PDGFRα-PE (clone aR1) (BD Biosciences), TRA-1-85-FITC/PE (clone TRA-1-85), and APLNR-APC (clone 72133) (R&D Systems). Control staining with appropriate isotype-matched mouse monoclonal antibodies controls was included to establish threshold for positive staining and subset gating. Sorting was conducted on a FACS Aria (BD), as described previously (Choi et al., 2012; Vodyanik and Slukvin, 2007).

qPCR Assay

Cells were differentiated for 3 and 4 days and sorted on a FACSAria for the populations of interest from each day. RNA was collected using quick-RNA MiniPrep (Zymo Research) and quantified on a NanoDrop (GE Healthcare). Equal amounts of RNA was used for cDNA synthesis using SuperScript III First-Strand Synthesis System (Life Technologies). qPCR was conducted using Platinum SYBR Green qPCR SuperMix (Life Technologies) and primers sequences are listed below.

Gene	Accession No.	Primer Sequence	Amplicon length
ETV2	NM_014209	F: TCTTTGAAGCGGTACCAGAG	108
		R: GGGACCTCGGTGGTTAGTT	
FOXF1	NM_001451	F: AGCCGAGCTGCAAGGCATC	180
		R: CAGCCTCACATCACGCAAGG	
GATA2	NM_001145661	F: GGCGCACAACTAGATGGAA	100
		R: GGTTGGCATAGTAGGGGTTG	
MIXL1	NM_031944	F: AGTCCAGGATCCAGGTATGG	123
		R: TTTCAGTTCCAGGAGCACAG	
T	NM_003181	F: GACAATTGGTCCAGCCTTG	92

		R: GGGTACTGACTGGAGCTGGT	
RPL13a	NM_012423	F: CCACCCTGGAGGAGAAGAGG<br>R: CGTGGGTCTTGAGGACCTCTC	139

The reactions were run on a Mastercycler RealPlex Thermal Cycler (Eppendorf) and the expression levels were calculated by minimal cycle threshold values (Ct) normalized to the reference expression of RPL13a. The qPCR products were run on an agarose gel and stained with ethidium bromide to confirm specificity of the primers.

TCR Rearrangement Assay

Genomic DNA was isolated using quick-gDNA MiniPrep (Zymo Research). TCRβ and TCRγ clonality was detected using a PCR amplification kit (Invivoscribe) and AmpliTaq Gold DNA polymerase (Applied Biosystems) as previously described (Hu et al., 2011). The PCR products were analysed using heteroduplex analysis on a 6% polyacrylamide gel stained with ethidium bromide.

Microarray analysis of mouse stromal cell lines

A mouse bone marrow stromal cell line, OP9, was obtained from Dr. Toru Nakano (Research Institute for Microbial Diseases, Osaka University, Japan), S17 was obtained from Dr. Kenneth Dorshkind (University of California, Los Angeles) and MS-5 was obtained from the German Tissue Culture Collection. Stromal cell lines were cultured as previously described (Vodyanik et al., 2005). DNA-free RNA was isolated using RiboPure RNA kit and treated with DNAse using TURBO DNAfree reagents (Ambion). All samples were processed at the Gene Expression Center of the Biotechnology Center at the University of Wisconsin, Madison. To analyze the total stromal cell lines, we used standard arrays containing 60-mer probes manufactured

by NimbleGen Systems (Madison, WI), which contains ~385,000 60-mer probes, covering approximately 36,000 Mus musculus genome transcripts (HG17, NCBI Build 35). Microarrays were hybridized and processed following previously published protocols (Yu et al., 2007). Gene expression raw data were extracted using NimbleScan software v2.1. Considering that the signal distribution of the RNA sample is distinct from that of the gDNA sample, the signal intensities from RNA channels in all eight arrays were normalized with Robust Multiple-chip Analysis (RMA) algorithm (Irizarry et al., 2003). Separately, the same normalization procedure was performed on those from the mouse gDNA samples. For a given gene, the median-adjusted ratio between its normalized intensity from the RNA channel and that from the gDNA channel was then calculated as follows: Ratio = intensity from RNA channel/(intensity from gDNA channel + median intensity of all genes from the gDNA channel). To find which genes were uniquely upregulated in overgrown OP9 cells, first we first selected genes that showed at least a 3 fold higher expression in overgrown OP9 cells versus semi-confluent OP9 cells, overgrown OP9 cells versus MS5 stromal cells and overgrown OP9 cells versus S17 stromal cells. Then, we selected the genes that were common in all three comparison groups.

Supplemental References (Experimental Procedures):

Choi, K.D., Vodyanik, M.A., Togarrati, P.P., Suknuntha, K., Kumar, A., Samarjeet, F., Probasco, M.D., Tian, S., Stewart, R., Thomson, J.A., *et al.* (2012). Identification of the hemogenic endothelial progenitor and its direct precursor in human pluripotent stem cell differentiation cultures. Cell Rep *2*, 553-567.

Hu, K., Yu, J., Suknuntha, K., Tian, S., Montgomery, K., Choi, K.D., Stewart, R., Thomson, J.A., and Slukvin, II (2011). Efficient generation of transgene-free induced pluripotent stem cells from normal and neoplastic bone marrow and cord blood mononuclear cells. Blood *117*, e109-119.

Irizarry, R.A., Hobbs, B., Collin, F., Beazer-Barclay, Y.D., Antonellis, K.J., Scherf, U., and Speed, T.P. (2003). Exploration, normalization, and summaries of high density oligonucleotide array probe level data. Biostatistics *4*, 249-264.

Vodyanik, M.A., Bork, J.A., Thomson, J.A., and Slukvin, II (2005). Human embryonic stem cell-derived CD34+ cells: efficient production in the coculture with OP9 stromal cells and analysis of lymphohematopoietic potential. Blood *105*, 617-626.

Vodyanik, M.A., and Slukvin, II (2007). Hematoendothelial differentiation of human embryonic stem cells. Curr Protoc Cell Biol *Chapter 23*, Unit 23 26.

Yu, J., Vodyanik, M.A., Smuga-Otto, K., Antosiewicz-Bourget, J., Frane, J.L., Tian, S., Nie, J., Jonsdottir, G.A., Ruotti, V., Stewart, R., *et al.* (2007). Induced pluripotent stem cell lines derived from human somatic cells. Science *318*, 1917-1920.

Supplementary Figures

Supplementary Figure S1

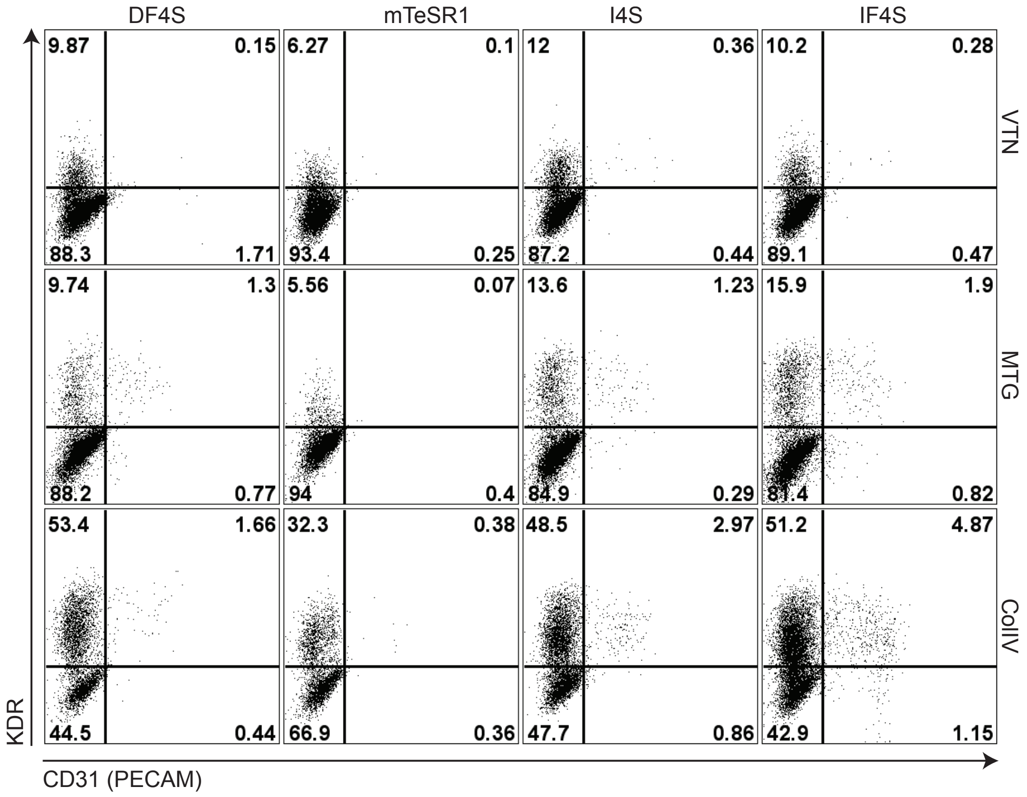

Supplementary Figure S1. Generation of KDR⁺CD31⁺ hematoendothelial progenitors in cultures using different basal media and matrix protein. mTeSR1 is mTeSR1 without FGF2 and TGFβ. DF4S is DMEM/F12-based media with 4 supplements: 64mg/L L-asorbic Acid 2-Phosphate $Mg2^{+}$ salt, 14μg/L sodium selenite, 10.6ng/L Holo-Transferrin, and 20mg/L Insulin. I4S is DF4S with IMDM-based media instead of DMEM/F12-based media, but with the 4 previously mentioned supplements. IF4S is DF4S with IMDM/F12-based media instead of DMEM/F12-based media, but with the 4 previously mentioned supplements. VTN is vitonectin matrix; MTG is Matrigel substrate; ColIV is Collagen IV matrix. Flow cytometry plots show percent of $CD31^{+}$ endothelial precursors of day 4 cells differentiated in each media supplemented with 50ng/ml FGF2, BMP4 and VEGF in hypoxia. Related to Figure 3.

Supplementary Figure S2

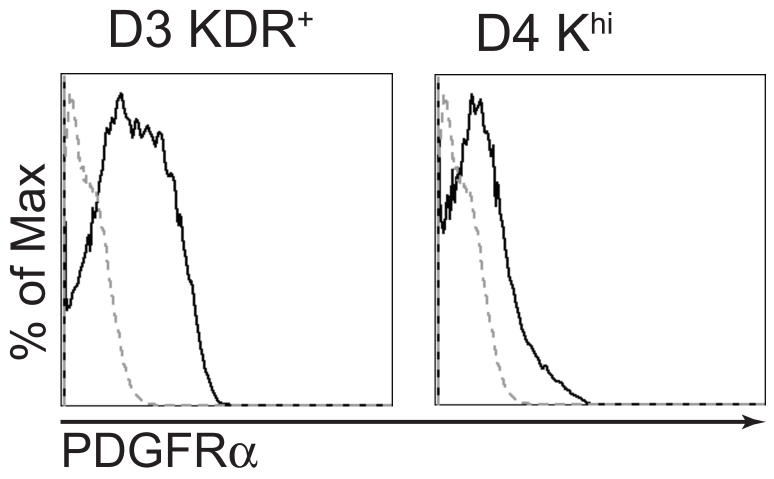

Supplementary Figure S2. Flow cytometry plot showing the expression of PDGFRα by gated day 3 KDR^{+} cells and by day 4 gated KDR^{high} (K^{hi}) cells. Related to Figure 3.

Supplementary Figure S3

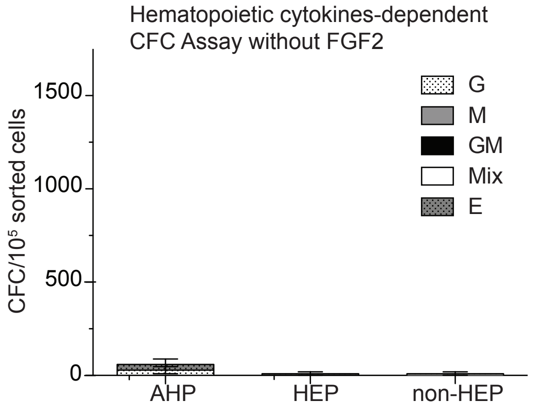

Supplementary Figure S3. CFC potential of isolated day 5 VEC^{+} subset in serum-free clonogenic medium containing hematopoietic cytokines without FGF2. AHP is VEC^{+}CD43/CD235^{+}CD73^{-} angiogenic hematopoietic progenitors, HEP is VEC^{+}CD43/CD235a^{-}CD73^{-} hemogenic endothelial progenitors, non-HEP VEC^{+}CD235a/CD43^{-}CD73^{+} non-hemogenic endothelial progenitors. Related to Figure 4.

Supplementary Figure S4

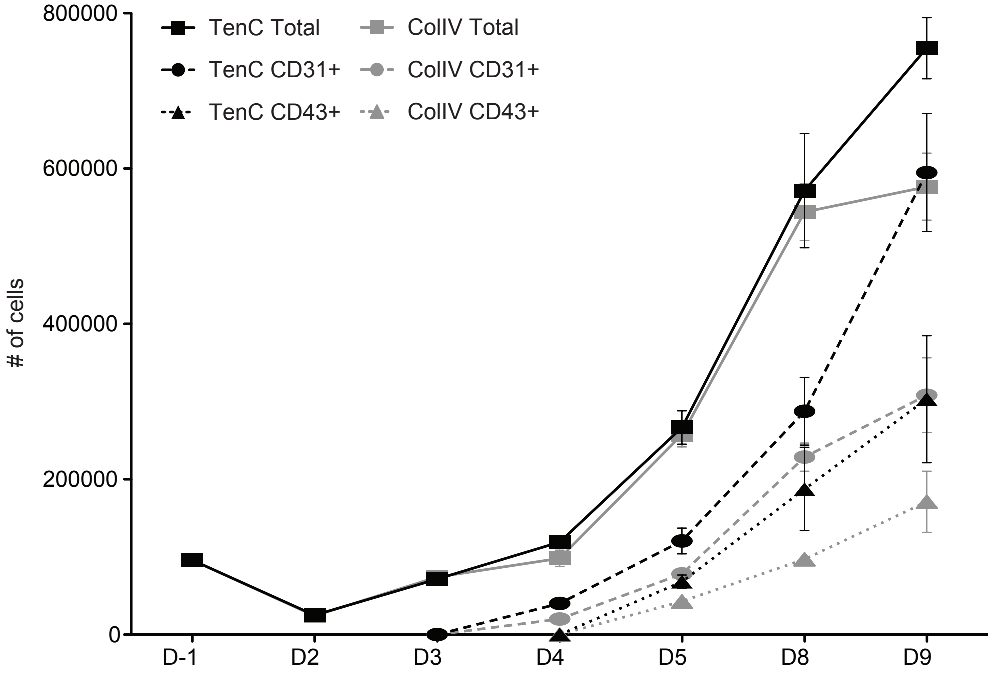

Supplementary Figure S4. Total number of cells generated from starting from day -1 when cells are plated on either TenC or ColIV, up to day 9 of differentiation. The numbers of $CD31^+$ and $CD43^+$ cells were calculated based on total number of cells times the percentage of positive cells based on flow cytometry. Results are mean ± SE of triplicates in one representative experiment. Related to Figure 4.

Supplementary Figure S5

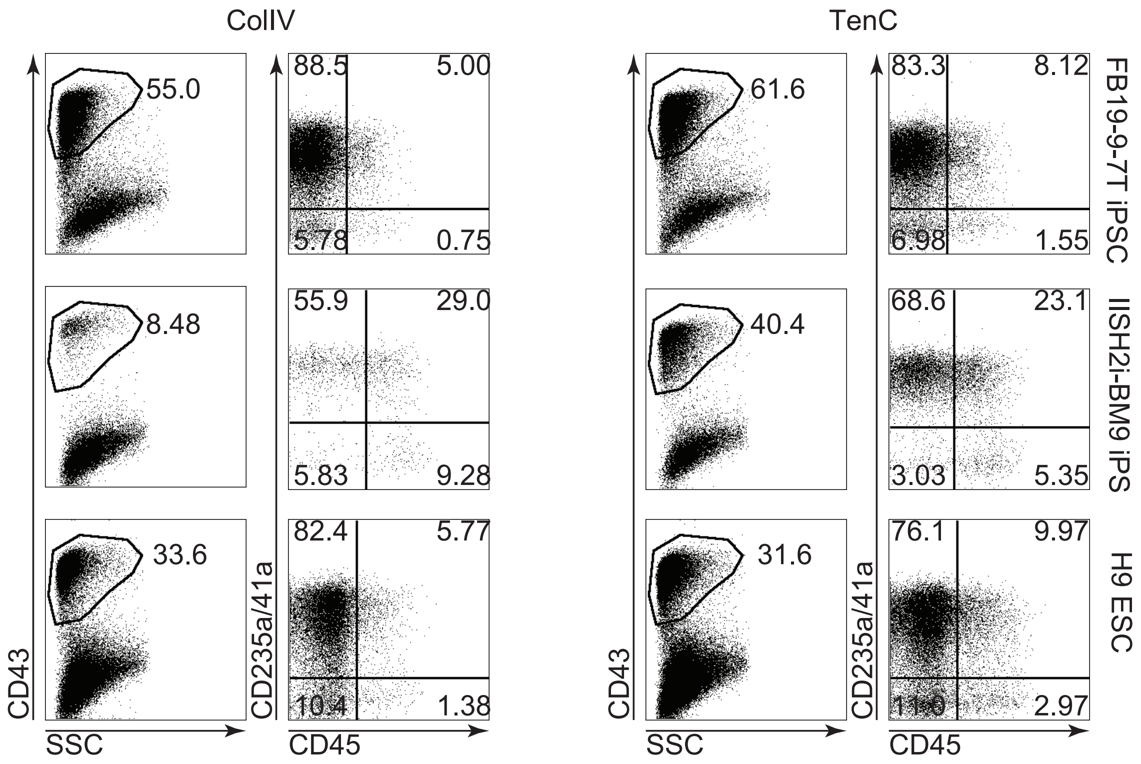

Supplemental Figure S5. Dot plots show the percentage of CD43⁺ cells and their subsets of DF19-9-7T human fibroblast iPSC line, IISH2i-BM9 human bone marrow-derived iPSC line, and H9 human ESC line differentiated for 8 days on either ColIV or TenC. Related to Figure 5.

Supplementary Table S1. Complete Content of IF9S medium.

IF9S medium	Amount	Unit/L
**Inorganic Salts**		
Calcium Chloride	99.3	mg
Cupric Sulfate • 5H2O	0.00125	mg
Ferrous Sulfate • 7H2O	0.417	mg
Magnesium Chloride • 6H2O	28.8	mg
Magnesium Sulfate (anhydrous)	48.835	mg
Potassium Chloride	277	mg
Potassium Nitrate	0.038	mg
Sodium Bicarbonate	2100	mg
Sodium Chloride	6052	mg
Sodium Phosphate Dibasic (anhydrous)	71.02	mg
Sodium Phosphate Monobasic (anhydrous)	54.5	mg
sodium selenite	0.017	mg
Zinc Sulfate • 7H2O	0.4315	mg
**Amino Acids**		
glycin	26.255	mg
L-alanyl-L-glutamine dipeptide	434.4	mg
L-alanine	25.9	mg
L-Arginine • HCl	160.7	mg
L-asparagine	21.705	mg
L-Aspartic acid	34.95	mg
L-Cysteine • HCl • H2O	17.5	mg
L-Cystine • 2HCl	45.62	mg
L-Glutamic Acid	59.55	mg
L-Glutamine	365	mg
L-Histidine • HCl • H2O	31.48	mg
L-Isoleucine	54.47	mg
L-Leucine	59.05	mg
L-Lysine • HCl	91.25	mg
L-Methionine	17.24	mg
L-Phenylalanine	35.48	mg
L-proline	48.75	mg
L-Serine	36.75	mg
L-Threonine	53.45	mg
L-Tryptophan	9.02	mg
L-Tyrosine • 2Na • 2H2O	55.785	mg
L-Valine	52.85	mg
**Vitamins**		
D-Biotin	0.01015	mg
Choline Chloride	8.98	mg
Folic Acid	2.66	mg
holo-transferrin	10.7	mg
myo-Inositol	12.6	mg
Niacinamide	2.0185	mg

D-Pantothenic Acid (hemicalcium)	2.24	mg
Pyridoxal • HCl	2	mg
Pyridoxine • HCl	0.031	mg
Riboflavin	0.219	mg
thiamine	2.17	mg
Vitamin B12	0.6865	mg
**Other**		
D-Glucose	3151	mg
HEPES	2979	mg
Hypoxanthine	2.04	mg
Phenol Red • Na	8.65	mg
Putrescine • HCl	0.0805	mg
DL-Thioctic Acid	0.105	mg
Pyruvic Acid • Na	110	mg
Thymidine	0.365	mg
1-thioglycerol	40	ul
polyvinyl alcohol	10	g
insulin	20	mg
L-ascorbic acid 2-phosphate magnesium salt	64	mg
**Lipids**		
arachidonic acid	0.004	mg
cholesterol	0.44	mg
DL-alpha-Tocopherol Acetate	0.14	mg
Linoleic Acid	0.0956	mg
Linolenic Acid	0.02	mg
Myristic Acid	0.02	mg
Oleic Acid	0.02	mg
Palmitic Acid	0.02	mg
Palmitoleic Acid	0.02	mg
Pluronic F-68	180	mg
Stearic Acid	0.02	mg
Tween-80	4.4	mg
