# Supplementary material for: Tenascin C Promotes Hematoendothelial Development and T Lymphoid Commitment from Human Pluripotent Stem Cells in Chemically Defined Conditions
Source: Stem Cell Reports. 2014 Oct 23;3(6):1073–84. doi: 10.1016/j.stemcr.2014.09.014 (PMC4263995; doi:10.1016/j.stemcr.2014.09.014)
Supplement: Document S2. Article plus Supplemental Information [file mmc2.pdf]

Stem Cell Reports
Article
OPEN ACCESS

Tenascin C Promotes Hematoendothelial Development and T Lymphoid Commitment from Human Pluripotent Stem Cells in Chemically Defined Conditions

Gene Uenishi,[1] Derek Theisen,[2] Jeong-Hee Lee,[2] Akhilesh Kumar,[2] Matt Raymond,[2] Maxim Vodyanik,[2] Scott Swanson,[3] Ron Stewart,[3] James Thomson,[3,4,5] and Igor Slukvin[1,2,*]
[1]Department of Pathology and Laboratory Medicine, University of Wisconsin, Madison, WI 53792, USA
[2]Wisconsin National Primate Research Center, University of Wisconsin, Madison, WI 53715, USA
[3]Morgridge Institute for Research, Madison, WI 53707, USA
[4]Department of Cell and Regenerative Biology, School of Medicine and Public Health, University of Wisconsin, Madison, WI 53707, USA
[5]Department of Molecular, Cellular & Developmental Biology, University of California, Santa Barbara, Santa Barbara, CA 93106, USA
*Correspondence: islukvin@wisc.edu
http://dx.doi.org/10.1016/j.stemcr.2014.09.014
This is an open access article under the CC BY license (http://creativecommons.org/licenses/by/3.0/).

SUMMARY

The recent identification of hemogenic endothelium (HE) in human pluripotent stem cell (hPSC) cultures presents opportunities to investigate signaling pathways that are essential for blood development from endothelium and provides an exploratory platform for de novo generation of hematopoietic stem cells (HSCs). However, the use of poorly defined human or animal components limits the utility of the current differentiation systems for studying specific growth factors required for HE induction and manufacturing clinical-grade therapeutic blood cells. Here, we identified chemically defined conditions required to produce HE from hPSCs growing in Essential 8 (E8) medium and showed that Tenascin C (TenC), an extracellular matrix protein associated with HSC niches, strongly promotes HE and definitive hematopoiesis in this system. hPSCs differentiated in chemically defined conditions undergo stages of development similar to those previously described in hPSCs cocultured on OP9 feeders, including the formation of VE-Cadherin^{+}CD73^{-}CD235a/CD43^{-} HE and hematopoietic progenitors with myeloid and T lymphoid potential.

INTRODUCTION

In the embryo, hemogenic endothelium (HE) has been identified as an immediate direct precursor of hematopoietic progenitors and hematopoietic stem cells (HSCs) (Bertrand et al., 2010; Boisset et al., 2010; Jaffredo et al., 2000; Kissa and Herbomel, 2010; Zovein et al., 2008). Thus, the ability to produce HE from human pluripotent stem cells (hPSCs) is considered a critical step toward the de novo generation of blood progenitors and stem cells. The recent identification and characterization of HE in hPSC cultures by our lab and others have provided a platform for investigating pathways that control HE formation and subsequent HSC specification (Choi et al., 2012; Kennedy et al., 2012; Rafii et al., 2013). However, the use of xenogeneic or allogeneic feeder cells, poorly defined serum and matrix proteins, or proprietary medium and supplements of undisclosed chemical composition limits the utility of the current differentiation systems for studying factors that are essential for HE development and specification. Here, after plating hPSCs from a single-cell suspension in a completely chemically defined medium that was free of serum components and xenogeneic proteins, we identified a set of factors and matrix proteins that are capable of supporting hematopoietic differentiation. Importantly, we showed the critical role of the HSC niche matrix component Tenascin C (TenC) in supporting the development of hematoendothelial and T lymphoid cells from hPSCs.

In our previous studies (Choi et al., 2012; Vodyanik et al., 2006, 2010), we identified distinct stages of hematoendothelial development following hPSC differentiation in coculture with OP9 (Figure 1). Plating hPSCs onto OP9 stromal cells induces the formation of primitive streak and mesodermal cells that can be detected based on the expression of apelin receptor (APLNR) and the absence of endothelial (CD31 and VE-cadherin [VEC]), endothelial/mesenchymal (CD73 and CD105), and hematopoietic (CD43 and CD45) cell-surface markers, i.e., by the ^{EMH}lin^{-} phenotype (Choi et al., 2012; Vodyanik et al., 2010). The early ^{EMH}lin^{-}APLNR^{+} cells that appear in OP9 coculture on day 2 of differentiation express primitive posterior mesoderm (PM) genes (*T*, *MIXL1*, *FOXF1*, and *GATA2*) and display the APLNR^{+}PDGFRα^{+}KDR^{+} phenotype (hereafter referred to as A^{+}P^{+} cells). These cells possess mesenchymoangioblast (MB) potential, i.e., the potential to form colonies with the capacity to differentiate into mesenchymal stem cells (MSC) and endothelial cells. On day 3 of differentiation, A^{+}P^{+} cells acquire blast (BL)-CFC or hemangioblast (HB) potential (Vodyanik et al., 2010). With advanced maturation, ^{EMH}lin^{-}APLNR^{+} mesodermal cells lose BL-CFC activity, upregulate KDR, and downregulate PDGFRα, i.e., they acquire the hematovascular mesodermal precursor (HVMP) phenotype,

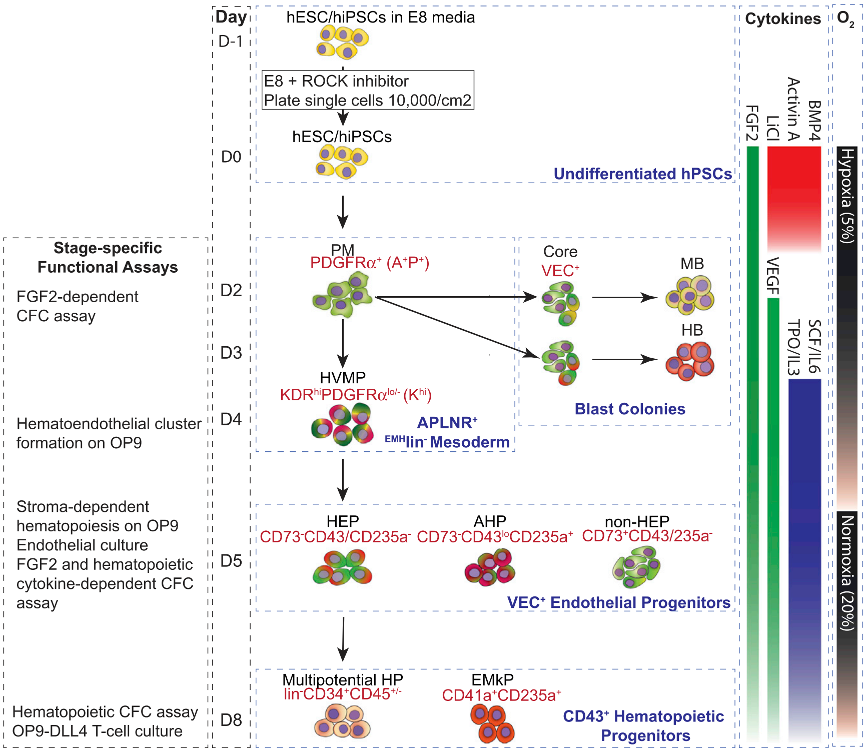

Figure 1. Schematic Diagram of Hematopoietic Differentiation and the Specific Markers and Functional Assays Used to Identify Each Stage of Development

The main cell subsets observed in previous differentiation studies using coculture with OP9 feeders (Choi et al., 2012; Vodyanik et al., 2006, 2010), and the current chemically defined cultures using ColIV and TenC matrices are shown. PM, primitive posterior mesoderm; HVMP, hematovascular mesodermal precursor; HEP, hemogenic endothelial progenitors; AHP, angiogenic hematopoietic progenitors; non-HEP, non-hemogenic endothelial progenitors; HP, hematopoietic progenitors; EMkP, erythromegakaryocytic progenitors. $^{EMH}lin^{-}$ indicates lack of expression of endothelial (CD31 and VEC), endothelial/mesenchymal (CD73 and CD105), and hematopoietic (CD43 and CD45) markers. lin^{-} indicates lack of expression of markers associated with hematopoietic lineages. MB, mesenchymoangioblast; HB, hemangioblast.

$^{EMH}lin^{-}KDR^{hi}APLNR^{+}PDGFR\alpha^{lo/-}$ (hereafter referred to as K^{hi} cells). K^{hi} HVMP cells downregulate the primitive streak genes *T* and *MIXL1*, and upregulate genes associated with lateral plate and hematovascular mesoderm development (*FOXF1*, *ETV2*, and *GATA2*). K^{hi} HVMPs are highly enriched in cells with the potential to form hematoendothelial clusters on OP9 (Choi et al., 2012). The first endothelial cells that coexpress VEC and CD31 emerge from K^{hi} mesodermal cells by day 4 of differentiation. The emerging VEC^{+} cells represent a heterogeneous population that includes $CD235a/CD43^{-}CD73^{+}$ nonhemogenic endothelial progenitors (non-HEPs) and CD235a/$CD43^{-}CD73^{-}$ hemogenic endothelial progenitors (HEPs) (Choi et al., 2012). HEPs lack hematopoietic CFC potential, but acquire it after coculture with stromal cells. The first hematopoietic cells that express CD43 emerge within the VEC^{+} cells on day 4–5 of differentiation. These cells express low levels of CD43 ($CD43^{lo}$) and coexpress CD235a, but lack CD41a expression, i.e., they have the phenotype $VEC^{+}CD43^{lo}235a^{+}41a^{-}$. Because these cells have the capacity to form hematopoietic colonies in the presence of FGF2 and hematopoietic cytokines, as well as to form a monolayer of endothelial cells on fibronectin, we designated them as angiogenic hematopoietic progenitors (AHPs). The $CD41a^{+}$ cells emerge within the $CD235a^{+}$ population. These $CD235a^{+}CD41a^{+}$ cells are highly enriched in erythro-megakaryocytic progenitors and lack endothelial potential. The progenitors with broad myelolymphoid potential and the $lin^{-}CD34^{+}CD43^{+}CD45^{-}$ phenotype can be detected in hPSC cultures shortly after the emergence of $CD235a^{+}CD41a^{+}$ cells. Acquisition of CD45 expression by lin^{-} cells is associated with progressive myeloid commitment (Vodyanik et al., 2006). In the present work, we demonstrated that a TenC-based, chemically defined system is able to generate all mesodermal and endothelial transitional stages and myelolymphoid progenitors that we observed using the serum- and OP9 feeder-based differentiation system described above. Because our differentiation system utilizes hPSCs growing in chemically defined xenogene-free Essential 8 (E8) medium on vitronectin (VTN) (Chen et al., 2011), it provides the opportunity to produce clinical-grade endothelial and myelolymphoid progenitors from hPSCs for therapeutic purposes.

RESULTS

IMDM/F12-Based Medium Is Essential for Efficient Differentiation of hPSCs into Hematoendothelial Lineages from a Single-Cell Suspension in 2D Culture

Previously, our lab developed an hPSC differentiation protocol for the efficient generation of hematopoietic progenitors using a coculture method on the mouse stromal cell

line OP9 (Vodyanik et al., 2005; Vodyanik and Slukvin, 2007). Although the OP9 system supports efficient generation of HE and multilineage hematopoietic progenitors (Figure 1), this system is very sensitive to variations in serum quality, stromal cell maintenance, and the size of the hPSC colonies and clumps used for differentiation (Choi et al., 2011; Vodyanik and Slukvin, 2007). Forming embryoid bodies (EBs) is another commonly used approach for inducing HE and hematopoietic progenitors from hPSCs (Kennedy et al., 2012; Ng et al., 2005, 2008; Wang et al., 2004). However, EB methods often rely on serum or undefined media and supplements, and also have significant drawbacks, such as asynchronous differentiation, high variability, and dependence on the initial clump size. Additionally, inconsistency in the quality of hPSCs caused by variations in the albumin batches used for hPSC maintenance may lead to variations in the efficiency of blood development.

To overcome these limitations, we set out to characterize chemically defined media and matrix proteins capable of supporting hematoendothelial differentiation without serum from a single-cell suspension of H1 human embryonic stem cells (hESCs) maintained in a completely defined xenogene-free system using E8 medium on VTN (Chen et al., 2011). First, we plated hESCs as single cells and allowed them to attach for 24 hr in E8 medium supplemented with 10 μM Rho kinase inhibitor on Matrigel (MTG), VTN, or Collagen IV (ColIV) in normoxia. Then, the medium was changed to either basal growth factor-free mTeSR1, E8 (DF4S), E8 with an IMDM base (I4S), or E8 with an IMDM/F12 base (IF4S) supplemented with human recombinant BMP4, FGF2, and VEGF factors, which are commonly used to induce blood formation from hPSCs (Pick et al., 2007; Salvagiotto et al., 2011). After 4 days of differentiation, the cell cultures were evaluated for the presence of $CD31^+$ cells, which coexpress KDR and VEC and are highly enriched in hematoendothelial progenitors (Choi et al., 2012). Flow-cytometric analysis showed that the cells that differentiated on ColIV-coated plates in IF4S differentiated most efficiently into $CD31^+$ hematoendothelial precursors (Figure S1 available online). Later, we found that the addition of polyvinylalcohol, nonessential amino acids (NEAA), GlutaMAX, chemically defined lipid concentrate, and monothioglycerol increased cell viability and differentiation efficiency (data not shown). The basal medium thus obtained is referred to as IF9S (IMDM/F12 plus nine supplements; see Table S1 for the complete composition of the medium). These results demonstrated that the selected medium and supplements made it possible to obtain hematoendothelial cells in a chemically defined, xenogene-free condition on ColIV matrix from hPSCs maintained in E8 medium.

Analysis of the Molecular Signatures of Hematopoiesis-Supporting Stromal Cell Lines Identified TenC as an Extracellular Matrix that Is Uniquely Expressed in OP9 Feeders with High Hematopoiesis-Inducing Potential

Previously, we showed that OP9 is superior to S17 and MS5 stromal cell lines for inducing hematopoietic differentiation (Vodyanik et al., 2005). We also found that day 8 overgrown OP9 cultures are superior to day 4 semiconfluent OP9 cultures for inducing hematopoietic CFCs, including multipotential GEMM-CFCs. The observation that the confluency of the stromal cells has an effect on differentiation efficiency suggested that an extracellular matrix influences hematoendothelial differentiation. In order to find the matrix protein(s) that is critical for the hematopoiesis-supporting activity of OP9, we performed molecular profiling of S17 and MS5 stromal cell lines with low hematopoiesis-inducing potential. In addition, we compared overgrown OP9 (day 8) with semiconfluent OP9 (day 4) monolayers. Transcriptome analysis revealed 21 genes that showed at least 3-fold higher expression in day 8 overgrown OP9 cells as compared with all other stromal cells (Figure 2A). These included genes encoding *Ptn* (pleiotrophin), a secreted regulator of HSC expansion and regeneration (Himburg et al., 2010); *Rspo3* (R-spondin 3), an important regulator of Wnt signaling and angioblast development (Kazanskaya et al., 2008); and the extracellular matrix protein *Postn* (periostin), which is required for B lymphopoiesis (Siewe et al., 2011). Interestingly, one the most highly upregulated genes in overconfluent OP9 was *Tnc* (TenC) (Figure 2B). TenC is expressed by mesenchymal cells underlying hematopoietic clusters in the aorta-gonado-mesonephros (AGM) region and is required for intraembryonic and postnatal hematopoiesis (Marshall et al., 1999; Nakamura-Ishizu et al., 2012; Ohta et al., 1998). It is also expressed in the bone marrow stem cell niche (Nakamura-Ishizu et al., 2012). Because of these unique properties, we tested whether TenC could support hematopoietic differentiation more effectively than ColIV.

TenC Facilitates the Development of Mesoderm and Hematoendothelial Precursors in Chemically Defined Cultures following Stage-Specific Treatment of FGF2, BMP4, Activin A, LiCl, VEGF, and Hematopoietic Cytokines

In previous studies, we identified the major stages of hematoendothelial development from hPSCs using the OP9 coculture system (Figure 1; Choi et al., 2012; Slukvin, 2013; Vodyanik et al., 2005, 2006, 2010). In order to reproduce the hematoendothelial development observed in OP9 coculture, we searched for the optimal combinations of morphogens, growth factors, and extracellular matrices to facilitate the stepwise progression of hPSC differentiation

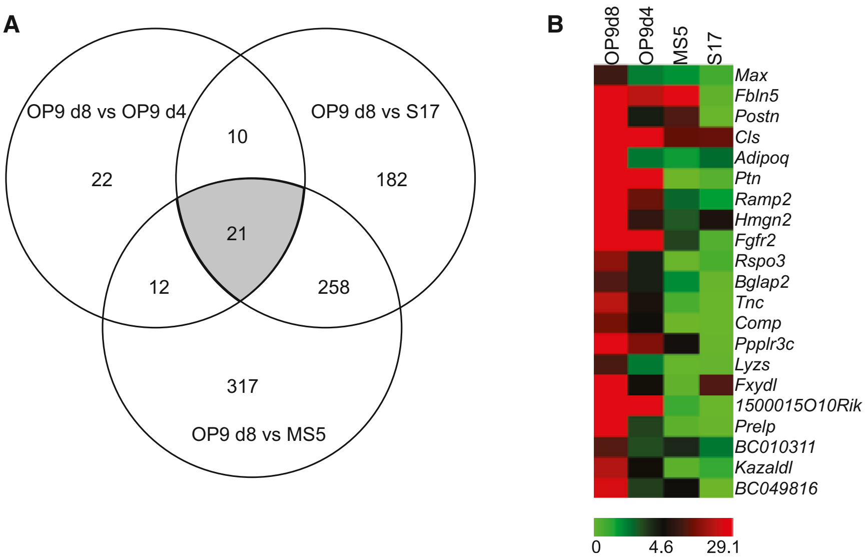

Figure 2. Comparison of Different Mouse Stromal Cell Lines that Support Hematopoietic Differentiation or Maintenance

(A) Venn diagram revealing the number of genes that were differentially expressed among the stromal cell lines. d4, day 4; d8, day 8.

(B) Heatmap of 21 genes uniquely upregulated in overconfluent (d8) OP9 stromal cell lines as compared with all other stromal cell lines (S17, MS5, and semiconfluent OP9 [d4]). TenC (*Tnc*) is one of the top differentially overexpressed genes in overgrown OP9 cells.

toward mesoderm, HE, and blood cells in chemically defined conditions.

During embryonic development, BMP4, Wnt, and TGFβ/Nodal/Activin A signaling pathways are critical for initiating primitive streak formation and subsequent mesoderm development (Gadue et al., 2005; Keller, 2005). It has been shown that the activation of these signaling pathways is essential to induce the expression of brachyury (T) and KDR (Flk-1, VEGFR2), and initiate mesodermal commitment of mouse PSCs and hPSCs (Cerdan et al., 2012; Kennedy et al., 2007; Nostro et al., 2008; Pearson et al., 2008; Pick et al., 2007; Salvagiotto et al., 2011). We found that high concentrations of BMP4 (50 ng/ml) combined with low concentrations of Activin A (15 ng/ml) and a supplement of LiCl (2 mM) consistently induced expression of the mesodermal surface markers APLNR, KDR, and PDGFRα after 2 days of culture of singularized hESCs on ColIV or TenC, as described above. However, these conditions poorly supported cell survival and required the addition of FGF2 and a hypoxic condition (5% O_2, 5% CO_2) to improve cell viability and output of mesodermal cells. Day 2 mesodermal cells that differentiated in these conditions expressed PDGFRα and APLNR surface markers, i.e., they became A^+P^+ cells and displayed MB colony-forming potential (Figures 3A and 3C), similar to what was observed for A^+P^+ mesodermal cells obtained from day 2 hPSCs differentiated in OP9 coculture (Vodyanik et al., 2010). After 2 days of differentiation, we found that only FGF2 and VEGF were sufficient for A^+P^+ mesoderm to acquire HB potential on day 3 of differentiation. Similarly to their counterparts generated in OP9 coculture, day 3 A^+P^+ cells that were generated in chemically defined conditions expressed *T* and *MIXL1* primitive streak genes at a high level, as well as *FOXF1* and *GATA2* lateral plate mesoderm genes (Figure 3C). The pattern of development was similar in cells cultured on ColIV and TenC. However, the TenC cultures produced significantly more A^+P^+ cells and MB and HB colonies (Figures 3A, 3B, and 3D).

We also found that only FGF2 and VEGF were sufficient to advance mesoderm specification toward a hematovascular fate, as signified by the increase of KDR and decrease of PDGFRα expression on day 4 of differentiation (Figures 3E and S2). Similarly to K^{hi} HVMP cells isolated form hPSC/OP9 cocultures, $KDR^{hi}CD31^-$ (K^{hi}) cells obtained in chemically defined conditions downregulated *T* and *MIXL1* primitive streak genes and upregulated the genes *FOXF1*, *ETV2*, and *GATA2*, which are associated with lateral plate and hematovascular mesoderm development (Figure 3C). Although day 4 differentiated cells lost HB potential (Figure 3D), K^{hi} cells were capable of forming hematoendothelial clusters when sorted and plated onto OP9 (Figure 3G). In contrast, KDR^{lo} (K^{lo}) cells only formed endothelial clusters with almost no hemogenic activity (Figure 3G). This is also consistent with differentiation in OP9 coculture (Choi et al., 2012). The percentage of K^{hi} HVMP cells was consistently higher in TenC cultures (Figure 3F).

Because the formation of HVMPs in hPSC/OP9 coculture was closely followed by the development of HE and blood progenitors, we supplemented our cultures with SCF, TPO, IL-6, and IL-3 hematopoietic cytokines in addition to VEGF and FGF2, starting from day 4 of differentiation. Although we noticed that the continuous treatment of cultures with FGF2 and VEGF was sufficient for induction of endothelial progenitors and hematopoietic specification, the addition of hematopoietic cytokines was essential to increase the output of these cells in chemically defined cultures. On day 5 of differentiation in these conditions, we observed the three major subsets of the VEC^+ population as

identified in a previous study (Choi et al., 2012): VEC$^+$CD235a/CD43$^-$CD73$^+$ (non-HEPs), VEC$^+$CD235a/CD43$^-$CD73$^-$ (HEPs), and VEC$^+$CD43/ CD235a$^+$ (AHPs) (Figures 4A and 4B). When these subsets were sorted and plated in endothelial conditions, they all formed a monolayer of VEC-expressing cells with the capacity to uptake AcLDL and form vascular tubes in the tube formation assay, consistent with OP9 coculture (Figure 4C). However, hematopoietic CFC potential was mostly restricted to the VEC$^+$CD43/CD235a$^+$ cells (Figure 4D). Importantly, similar to our previous finding with day 5 VEC $^+$ subsets generated in coculture with OP9, the hematopoietic CFC potential of VEC$^+$CD43/CD235a$^+$ cells was detected only in serum-free medium in the presence of FGF2 in addition to hematopoietic cytokines (Figures 4D and S3). This indicates that VEC$^+$CD43/CD235a$^+$ cells are essentially similar to the AHPs identified in hPSC/OP9 coculture (Choi et al., 2012). We previously defined HEPs as VEC$^+$CD43$^-$CD73$^-$ cells that lack hematopoietic CFC potential but are capable of acquiring it after culture on OP9 (Choi et al., 2012). To determine whether VEC$^+$CD43$^-$CD73$^-$ cells generated in completely chemically defined conditions are similar to HEPs produced in OP9 cocultures, we sorted the day 5 VEC$^+$ subpopulations and cultured them on OP9 as previously described (Choi et al., 2012). In these conditions, the HEPs formed both endothelial and hematopoietic cells with a large number of HE clusters, whereas AHPs formed predominantly hematopoietic cells with few endothelial cells and hematoendothelial clusters. VEC$^+$CD43$^-$CD73$^+$ cells formed exclusively endothelial clusters, consistent with the non-HEP phenotype (Figure 4C). Cultures that differentiated on TenC had a larger population of total CD31$^+$ and VEC$^+$ cells, and thus increased populations of HEPs, non-HEPs, and AHPs compared with cultures differentiated on ColIV (Figures 4A, 4B, and S4).

When numerous floating, round hematopoietic cells became visible in cultures on day 6, the hypoxic conditions were not necessary to sustain hematopoietic development. Therefore, from day 6 of differentiation, the cultures were transferred to a normoxic incubator (20% O_2, 5% CO_2). By day 8 of differentiation, the cultures continued to develop and expand the CD43$^+$ hematopoietic cells, which consisted of CD235a$^+$CD41a$^+$ cells enriched in erythro-megakaryocytic progenitors and CD235a/CD41a$^-$ CD43$^+$CD45$^{-/+}$ cells that expressed CD34 but lacked other lineage markers (lin$^-$) (Figure 5). Consistent with cells that differentiated on OP9, hematopoietic colony-forming potential was limited to the CD43$^+$ subpopulations (Figure 5C). CD43$^+$ hematopoietic progenitors were generated in significantly higher numbers on TenC compared with ColIV (Figures 5A, 5B, and S4). In addition, the GEMM-CFC potential was significantly greater in cultures on TenC compared with those on ColIV (Figure 5D).

TenC Is Superior to ColIV for Supporting Hematopoietic Differentiation from a Variety of hPSC Lines

Although we developed the differentiation protocol using H1 hESCs, we found that the chemically defined conditions described here also supported the formation of HE and blood progenitors from another hESC line (H9) and human induced pluripotent stem cells (hiPSCs) generated from fibroblasts or bone marrow mononuclear cells (Figure S5). Previously, we demonstrated that hiPSCs obtained through reprogramming of bone marrow mononuclear cell (BM) hiPSCs differentiated less efficiently into blood cells on OP9 feeders compared with fibroblast-derived (FB) hiPSCs (Hu et al., 2011). We reproduced that finding when we differentiated BM and FB iPSCs on ColIV. However, differentiation on TenC restored the hematopoietic differentiation potential of BM hiPSCs to the level seen with hESCs and FB hiPSCs (Figure S5), thereby confirming that TenC is superior to ColIV for promoting hematopoietic differentiation from hPSCs.

TenC Uniquely Supports Specification of T Lymphoid Progenitors from hPSCs

To find out whether our culture system supports the establishment of the definitive hematopoietic program from hPSCs, we analyzed the T cell potential of blood cells generated in our system as an indicator of definitive hematopoiesis (Kennedy et al., 2012). When we collected CD43$^+$ floating cells from day 9 differentiated cultures and replated them onto OP9 expressing DLL4 (OP9-DLL4), CD7$^+$CD5$^+$ lymphoid progenitors began to emerge by week 2 of coculture. By week 3, CD4$^+$CD8$^+$ double-positive T cells arose (Figure 6A). Interestingly, CD43$^+$ cells generated on both ColIV and TenC had the capacity to generate CD5$^+$CD7$^+$ lymphoid progenitors, although CD43$^+$ cells generated on ColIV had a significantly lower potential. However, progression toward CD4$^+$CD8$^+$ T lymphoid cells was consistently observed only from CD43$^+$ cells generated on TenC, and not from ColIV cultures (Figure 6B). To confirm T cell development, we analyzed the genomic DNA of the hematopoietic cells from OP9-DLL4 cultures for the presence of T cell receptor (TCR) rearrangements. This analysis demonstrated the presence of multiple PCR products of random V-J and D-J rearrangements at the β locus and V-J rearrangements at the γ locus, indicative of a polyclonal T lineage repertoire (Figures 6C and 6D). Overall, these findings signify that the extracellular matrix protein TenC is essential for supporting the generation of hematopoietic cells with myeloid and lymphoid potential from hPSCs in chemically defined conditions. However, we failed to obtain engraftment following transplantation TenC differentiated cells in immunocompromised mice (data not shown), which suggests that additional

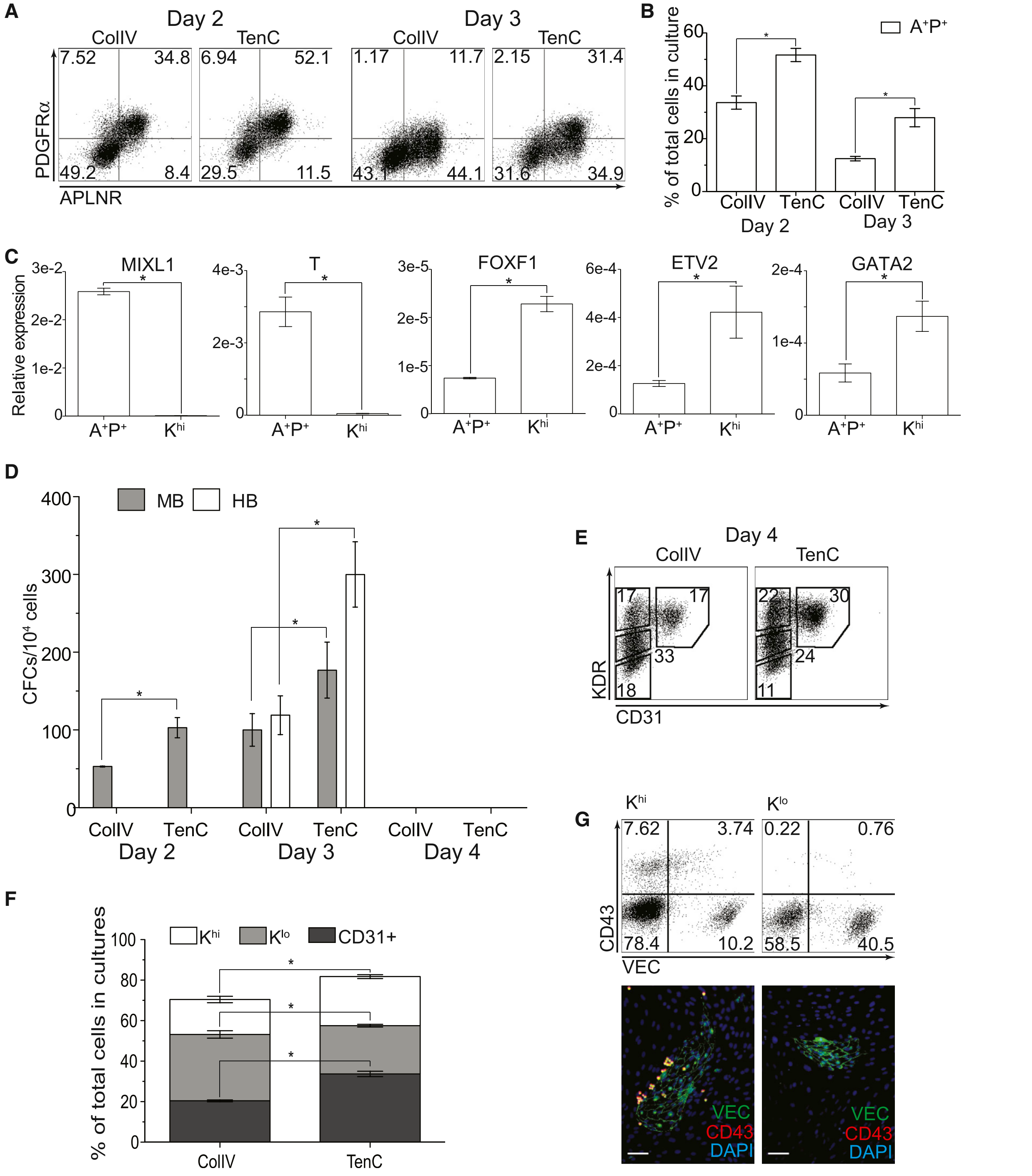

Figure 3. Mesodermal Development from H1 hESCs in Chemically Defined Conditions on ColIV and TenC

Cultures differentiated on ColIV versus TenC for 2, 3, and 4 days in chemically defined conditions.

(A and B) Flow-cytometry plots (A) and graphs (B) comparing the percentage of A^+P^+ primitive mesodermal population on days 2 and 3.

(C) Expression of mesoderm lineage genes measured by quantitative PCR and normalized to RPL13A, comparing day 3 P^+ cells and day 4 K^{hi} cells.

(D) Comparison of the MB/HB colony-forming potential of day 2, day 3, and day 4 cultures.

(E and F) Flow-cytometry plots (E) and graphs (F) comparing the percentage of $KDR^{hi}CD31^-$ (K^{hi}) HVMP, $CD31^+$, and $KDR^{lo}CD31^-$ (K^{lo}) populations on day 4 of differentiation.

(legend continued on next page)

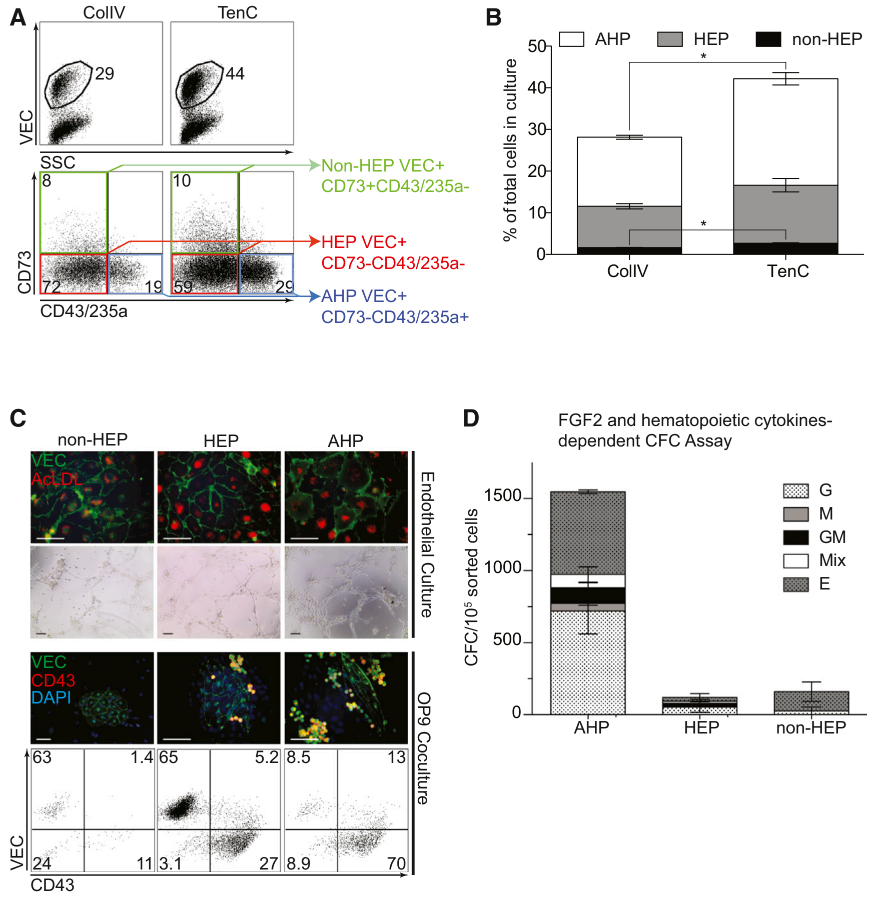

Figure 4. Major Subsets of VEC^{+} Cells Generated after 5 Days of Differentiation of H1 hESCs in Chemically Defined Conditions on ColIV and TenC

(A) Flow-cytometric analysis demonstrates major subsets of VEC^{+} progenitors generated after 5 days of hESC culture in chemically defined conditions on ColIV and TenC. Lower dot plots show VEC^{+}-gated cells.

(B) Percentages of VEC^{+} cells and subsets generated in ColIV and TenC cultures. Error bars are mean ± SE from at least three experiments (*$p < 0.01$).

(C) Endothelial and hematopoietic potential of day 5 VEC^{+} subsets. Progenitor subsets sorted and cultured either in endothelial conditions with subsequent tube formation assay or on OP9 with immunofluorescent and flow-cytometry results after 7 days. Dot plots show expression of VEC and CD43 in TRA-1-85^{+}-gated human cells. Scale bars, 100 μM.

(D) CFC potential of an isolated VEC^{+} subset in serum-free clonogenic medium containing hematopoietic cytokines and FGF2. Error bars are mean ± SE from three experiments (*$p < 0.01$). Scale bar represents 100 μm. VEC was visualized using a secondary antibody conjugated to DyLight 488, and CD43 was visualized using a secondary antibody conjugated to DyLight 594.

See also Figures S3 and S4.

maturation signals are required to activate the self-renewal program in hematoendothelial progenitors generated in our system.

DISCUSSION

During the last decade, significant progress has been made in achieving hematopoietic differentiation from hPSCs. Multiple protocols for hematopoietic differentiation have been developed and have made it possible to routinely produce blood cells for experimentation. However, generating HSCs with long-term reconstitution potential from hPSCs remains a significant challenge. Hematopoietic cells and HSCs arise from a specific subset of endothelium (HE) in the embryo (Bertrand et al., 2010; Boisset et al., 2010; Jaffredo et al., 2000; Kissa and Herbomel, 2010; Zovein et al., 2008). Therefore, the ability to interrogate the signaling pathways that induce HE specification and the endothelial-to-hematopoietic transition in a completely chemically defined environment is essential in order to identify the factors required for HSC specification. Although the original protocols for hematopoietic differentiation employed xenogeneic feeder cells and/or serum, several serum- and feeder-free systems for hematopoietic differentiation have been described recently (Ng et al., 2008; Salvagiotto et al., 2011; Smith et al., 2013; Wang et al., 2012). However, these protocols still require serum components (albumin) and it remains unclear whether these protocols reproduce the distinct waves of

(G) Hematopoietic and endothelial potential of K^{hi} and K^{lo} cells isolated from day 4 differentiated cells after coculture on OP9 for 7 days. Upper panels show flow cytometry of TRA-1-85^{+}-gated human cells and lower panels show immunofluorescence staining of cells from OP9 cocultures with K^{hi} and K^{lo} cells.

In (B)–(D) and (G), bars are mean ± SE from at least three experiments (*$p < 0.01$). Scale bar represents 100 μm. VEC was visualized using a secondary antibody conjugated to DyLight 488, and CD43 was visualized using a secondary antibody conjugated to DyLight 594. See also Figures S1 and S2.

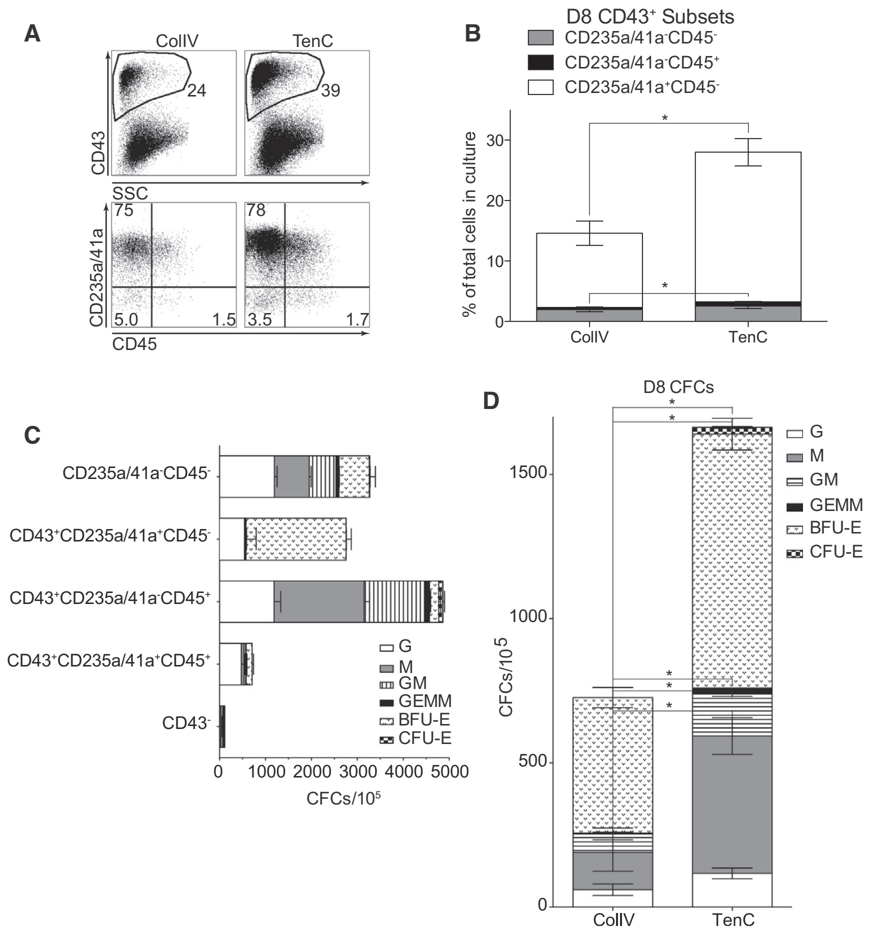

Figure 5. Major Subsets of CD43$^+$ Cells Generated after 8 Days of Differentiation of H1 hESCs in Chemically Defined Conditions on ColIV and TenC

(A) Flow-cytometry analysis shows major subsets of CD43$^+$ cells generated in cultures on ColIV and TenC. Lower dot plots show CD43$^+$-gated cells.

(B) Cultures on TenC produce more CD43+ cells.

(C) Hematopoietic CFC potential is limited to the CD43+ subpopulations.

(D) Cultures differentiated on TenC produce more CFCs than cultures differentiated on ColIV.

In (B)–(D), error bars are mean ± SE from at least three experiments (*$p < 0.01$). See also Figure S5.

hematopoiesis, including the generation of HE with definitive lymphomyeloid potential, observed in the original differentiation systems. Recently, Kennedy et al. (2012) developed a feeder- and stroma-free condition for EB-based hematopoietic differentiation in a proprietary medium with undisclosed nutrient supplements from hPSCs expanded on mouse embryonic fibroblasts. These conditions reproduced primitive and definitive waves of hematopoiesis and generated HE with T lymphoid potential. Here, we developed a protocol that enables the efficient production of blood cells in completely chemically defined conditions, free of serum and xenogeneic proteins, from a single-cell suspension of hPSCs maintained in chemically defined E8 medium (Chen et al., 2011). Our protocol eliminates the variability associated with animal- or human-sourced albumins, xenogenic matrices, clump size variation, and asynchronous differentiation observed in EB systems. It also reproduces the typical waves of hematopoiesis, including the formation of HE and definitive hematopoietic progenitors, observed in hPSCs differentiated on OP9. Importantly, based on molecular profiling of OP9 and stromal cell lines with different hematopoiesis-inducing activity, we found that the TenC matrix protein, which is uniquely expressed in OP9 with robust hemato-inducing potential, strongly promotes hematoendothelial and T lymphoid development from hPSCs. TenC is a disulfide-linked hexameric glycoprotein that is mainly expressed during embryonic development. Although TenC mostly disappears in adult organisms, its expression is upregulated during wound repair, neovascularization, neoplasia (Hsia and Schwarzbauer, 2005), and limb regeneration (Stewart et al., 2013). TenC is found in adult bone marrow, where it is expressed predominantly in the endosteal region (Klein et al., 1993; Soini et al., 1993). TenC supports the proliferation of bone marrow hematopoietic cells (Seiffert et al., 1998) and erythropoiesis (Seki et al., 2006). TenC-deficient mice were shown to have lower bone marrow CFC potential (Ohta et al., 1998), failed to reconstitute hematopoiesis after bone marrow ablation, and showed a reduced ability to support engraftment of wild-type HSCs (Nakamura-Ishizu et al., 2012). In addition, TenC is expressed in the thymus (Hemesath and Stefansson, 1994) and plays an important role in T cell development, as evidenced by decreased T lymphoid progenitors in the

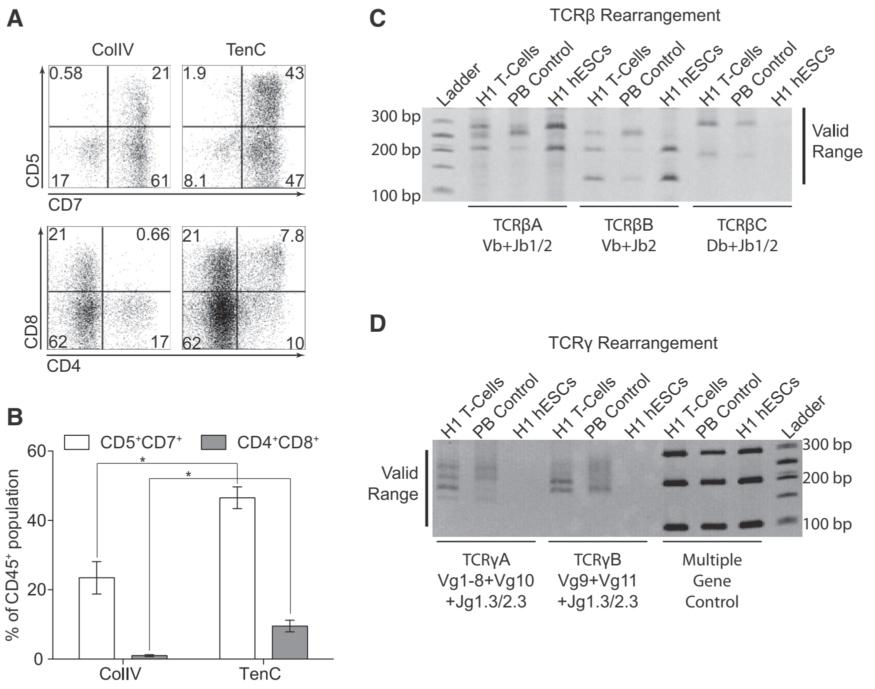

Figure 6. T Cell Potential of Hematopoietic Cells Collected from H1 hESC Cultures Differentiated for 9 Days in Chemically Defined Conditions on either ColIV or TenC

(A and B) Flow-cytometry analysis (A) and percentages (B) of cells collected under ColIV or TenC conditions after culture on OP9-DLL4 for 3 weeks. Error bars are mean + SE from at least three experiments (*p < 0.01).

(C and D) Analysis for TCR rearrangement by genomic PCR. H1 T cells are T cells derived from differentiating H1 hESCs on TenC, PB control is peripheral blood (positive control), and H1 hESCs are undifferentiated H1 hESCs (negative control).

thymus and an increased proportion of T cells in the bone marrow of TenC-deficient mice (Ellis et al., 2013). Interestingly, high levels of TenC expression were also detected in the human and chicken AGM region (Anstrom and Tucker, 1996; Marshall et al., 1999), the site where the first HSCs emerge, and in hematopoietic sites of human fetal liver (Papadopoulos et al., 2004). Because TenC expression is highly enriched in the subaortic mesenchyme directly underneath hematopoietic clusters, it was suggested that TenC plays a pivotal role in HSC development during embryogenesis (Marshall et al., 1999). TenC is also involved in the regulation of angiogenesis and cardiac endothelial progenitors (Ballard et al., 2006). Our studies demonstrated the superior properties of TenC for promoting hematoendothelial development from hPSCs. The positive effect of TenC was obvious at all stages of differentiation, including the enhancement of hematovascular mesoderm, HE, and $CD43^+$ hematopoietic progenitors. Importantly, TenC was able to support the development of definitive hematopoietic cells with T lymphoid potential, whereas we were not able to obtain such cells in cultures on ColIV. The TenC molecule is composed of an amino-terminal oligomerization region followed by heptad repeats, EGF-like and fibronectin type III repeats, and a fibrinogen globe (Hsia and Schwarzbauer, 2005). Each of these domains interacts with different surface receptors, including integrins $\alpha 9\beta 1$, $\alpha v\beta 3$, and $\alpha v\beta 6$, and toll-like receptor 4 (TLR-4) (Midwood et al., 2011). It is believed that the effect and interaction of TenC with cells requires the integrated action of multiple domains (Fischer et al., 1997), although several unique mitogenic domains capable of inducing the proliferation of hematopoietic cells were identified within this molecule (Seiffert et al., 1998). The interaction of TenC with $\alpha 9\beta 1$ integrin plays a central role in TenC-mediated expansion of hematopoietic stem and progenitor cells (Nakamura-Ishizu et al., 2012) and may be required for normal T cell development (Ellis et al., 2013). Several signaling mechanisms implicated in cell interaction with TenC have been identified, including the suppression of fibronectin-activated focal adhesion kinase signaling, Rho-mediated kinase signaling, and stimulation of Wnt signaling pathways (reviewed in Orend, 2005). Further studies to identify the mechanism of TenC signaling on hPSCs and their hematopoietic derivatives would help elucidate the role of this matrix protein during development. It is also important to determine the developmental stages that are most affected by TenC and clarify whether TenC simply enhances the expansion of hemogenic populations or promotes hematoendothelial commitment.

In summary, the findings presented here identify the TenC matrix protein, as well as completely chemically defined conditions that are free of serum/serum components and animal proteins and are capable of supporting the scalable production of HE and definitive blood cells from hPSCs. This differentiation system will enable the precise interrogation of signaling molecules implicated in hematopoietic differentiation and provides a platform for producing cGMP-grade blood cells for clinical application.

EXPERIMENTAL PROCEDURES

hPSC Maintenance

hPSCs, WA01 (H1) and WA09 (H9) hESCs, the DF19-9-7T human fibroblast iPSC line, and the IISH2i-BM9 bone marrow-derived iPSC line (WiCell, Madison, WI) were maintained on VTN or MTG in E8 medium supplemented with FGF2 and TGFβ (Peprotech). Cells were passaged when they reached 80% confluency using 0.5 mM EDTA in PBS. The cells were maintained in normoxic conditions with 5% CO_2.

hPSC Differentiation

Single-cell suspensions of hPSCs were obtained by treating the hPSC cultures at 80% confluency with 1× TrypLE (Life Technologies). Single cells were plated at an optimized density ranging from 5,000 cells/cm^2 to 15,000 cells/cm^2 (depending on the cell line) onto six-well plates coated with 0.5 μg/cm^2 of ColIV (Sigma-Aldrich) or 0.5 μg/cm^2 TenC (Millipore) in E8 medium supplemented with 10 μM Rho kinase inhibitor (Tocris Y-27632). After 24 hr (day 0), the medium was changed to IF9S medium (see Table S1 for the complete composition of the medium) supplemented with 50 ng/ml BMP4 (Peprotech), 15 ng/ml Activin A (Peprotech), 50 ng/ml FGF2 (Miltenyi Biotech), 2 mM LiCl (Sigma), and, on occasion, 1 μM Rho kinase inhibitor to increase cell viability. On day 2, the medium was changed to IF9S medium supplemented with 50 ng/ml FGF2 and 50 ng/ml VEGF. On day 4, the medium was changed to IF9S medium supplemented with 50 ng/ml FGF2, VEGF, TPO, SCF, IL-6, and 10 ng/ml IL-3. On day 6, additional IF9S medium supplemented with the same six factors were added to the cultures without aspirating the old medium. IF9S (IMDM/F12 with nine supplements) was made in-house with the following components: 50% IMDM and 50% F12 (Life Technologies) supplemented with 64 mg/l L-ascorbic acid 2-phosphate Mg^{2+} salt (Sigma-Aldrich), 40 μl/l monothioglycerol (Sigma-Aldrich), 8.4 μg/l additional sodium selenite (Sigma-Aldrich), 10 g/l polyvinyl alcohol (Sigma-Aldrich), 1× GlutaMAX (Life Technologies), 1× nonessential amino acids (Life Technologies), 0.1× chemically defined lipid concentrate (Life Technologies), 10.6 mg/l Holo-Transferrin (Sigma-Alrich), and 20 mg/l insulin (Sigma-Aldrich). Differentiation was conducted in a hypoxic condition from day 0 to day 5, and then in a normoxic condition from day 6 to day 9 (Figure 1). The 1× TrypLE was used to dissociate and collect cells for analysis.

MB, HB, and Hematopoietic CFC Assays

MB and HB were detected as described previously (Vodyanik et al., 2010). Hematopoietic CFCs were detected using serum-containing H4436 Methocult (Stem Cell Technologies) or serum-free H4236 Methocult with added FGF2 (20 ng/ml), SCF (20 ng/ml), IL-3 (10 ng/ml), IL-6 (10 ng/ml), and EPO (2 U/ml) as described previously (Choi et al., 2012).

Assessment of the Hematoendothelial Potential of Differentiated hPSCs

Sorted day 4 or day 5 cultures were plated on a confluent layer of OP9 cells in α-MEM (GIBCO) supplemented with 10% FBS (Hyclone) supplemented with 100 μM monothioglycerol, 50 μg/ml ascorbic acid, 50 ng/ml SCF, TPO, IL-6, and 10 ng/ml IL-3 at a density of 5,000 cells/well of a six-well plate as described previously (Choi et al., 2012). Cultures were analyzed 4–7 days later by immunofluorescent staining or by flow cytometry (Choi et al., 2012).

T Cell Differentiation of Day 9 Cultures

The OP9 cell line expressing human DLL4 (OP9-DLL4) was established by using lentivirus expressing human DLL4 under the EF1a promoter. After hPSC differentiation for 9 days, the floating $CD43^+$ cells were collected; strained through a 70 μm cell strainer (BD Biosciences); resuspended in T cell differentiation medium consisting of α-MEM (GIBCO) supplemented with 20% FBS (Hyclone), IL7 (5 ng/ml), FLT3L (5 ng/ml), and SCF (10 ng/ml); and cultured on OP9-DLL4. After 4 days, the cells were harvested using a collagenase IV (GIBCO) solution (1 mg/ml in DMEM/F12; GIBCO) and 1× TrypLE (Life Technologies), and passaged onto a fresh layer of OP9-DLL4. After 3 days, the cells were passaged again. Subsequent passages were conducted every 7 days for up to 4 weeks, and then floating cells were collected for flow analysis and genomic DNA extraction for TCR rearrangement assay.

Statistical Analysis

Statistical analysis was performed using Microsoft Excel. Data obtained from multiple experiments were reported as the mean ± SE. A two-tailed Student's t test was used to compare two groups. Differences were considered significant when $p < 0.01$.

ACCESSION NUMBERS

The microarray data reported in this work have been deposited in the Gene Expression Omnibus under accession number GSE61580.

SUPPLEMENTAL INFORMATION

Supplemental Information includes Supplemental Experimental Procedures, five figures, and one table and can be found with this article online at http://dx.doi.org/10.1016/j.stemcr.2014.09.014.

AUTHOR CONTRIBUTIONS

G.U. designed, conducted, and analyzed experiments; interpreted experimental data; made figures; and contributed to writing of the paper. D.T. conducted and analyzed experiments. J.L. generated OP9-DLL4 cells. J.L. and A.K. performed T cell differentiation. M.R. performed quantitative PCR assays. M.V. prepared different mouse stromal cell lines for molecular analysis and analyzed their hematopoiesis-inducing properties. R.S. and S.S. performed bioinformatics analysis of microarray data. J.T. contributed to concept development and directed molecular profiling studies. I.S. developed the concept, led and supervised all aspects of the studies, analyzed and interpreted data, and wrote the paper.

ACKNOWLEDGMENTS

We thank Mitchell Probasco for cell sorting, Katherine Jensen for consultation regarding the statistical analysis, and Dr. T. Nakano and K. Dorshkind for providing OP9 and S17 bone marrow stromal

cell lines. This work was supported by funds from the NIH (R01HL116221, U01HL099773, and P51 RR000167) and The Charlotte Geyer Foundation. J.A.T. owns stock in, serves on the Board of Directors of, and serves as Chief Scientific Officer of Cellular Dynamics International. I.S. is a founding shareholder and consultant for Cellular Dynamics International and Cynata. M.V. is a founding shareholder and consultant for Cynata.

Received: July 11, 2014
Revised: September 22, 2014
Accepted: September 23, 2014
Published: October 23, 2014

REFERENCES

Anstrom, K.K., and Tucker, R.P. (1996). Tenascin-C lines the migratory pathways of avian primordial germ cells and hematopoietic progenitor cells. Dev. Dyn. *206*, 437–446.

Ballard, V.L., Sharma, A., Duignan, I., Holm, J.M., Chin, A., Choi, R., Hajjar, K.A., Wong, S.C., and Edelberg, J.M. (2006). Vascular tenascin-C regulates cardiac endothelial phenotype and neovascularization. FASEB J. *20*, 717–719.

Bertrand, J.Y., Chi, N.C., Santoso, B., Teng, S., Stainier, D.Y., and Traver, D. (2010). Haematopoietic stem cells derive directly from aortic endothelium during development. Nature *464*, 108–111.

Boisset, J.C., van Cappellen, W., Andrieu-Soler, C., Galjart, N., Dzierzak, E., and Robin, C. (2010). In vivo imaging of haematopoietic cells emerging from the mouse aortic endothelium. Nature *464*, 116–120.

Cerdan, C., McIntyre, B.A., Mechael, R., Levadoux-Martin, M., Yang, J., Lee, J.B., and Bhatia, M. (2012). Activin A promotes hematopoietic fated mesoderm development through upregulation of brachyury in human embryonic stem cells. Stem Cells Dev. *21*, 2866–2877.

Chen, G., Gulbranson, D.R., Hou, Z., Bolin, J.M., Ruotti, V., Probasco, M.D., Smuga-Otto, K., Howden, S.E., Diol, N.R., Propson, N.E., et al. (2011). Chemically defined conditions for human iPSC derivation and culture. Nat. Methods *8*, 424–429.

Choi, K.D., Vodyanik, M., and Slukvin, I.I. (2011). Hematopoietic differentiation and production of mature myeloid cells from human pluripotent stem cells. Nat. Protoc. *6*, 296–313.

Choi, K.D., Vodyanik, M.A., Togarrati, P.P., Suknuntha, K., Kumar, A., Samarjeet, F., Probasco, M.D., Tian, S., Stewart, R., Thomson, J.A., and Slukvin, I.I. (2012). Identification of the hemogenic endothelial progenitor and its direct precursor in human pluripotent stem cell differentiation cultures. Cell Rep *2*, 553–567.

Ellis, S.L., Heazlewood, S.Y., Williams, B., Reitsma, A.J., Grassinger, J., Borg, J., Heazlewood, C.K., Chidgey, A.P., and Nilsson, S.K. (2013). The role of Tenascin C in the lymphoid progenitor cell niche. Exp. Hematol. *41*, 1050–1061.

Fischer, D., Brown-Lüdi, M., Schulthess, T., and Chiquet-Ehrismann, R. (1997). Concerted action of tenascin-C domains in cell adhesion, anti-adhesion and promotion of neurite outgrowth. J. Cell Sci. *110*, 1513–1522.

Gadue, P., Huber, T.L., Nostro, M.C., Kattman, S., and Keller, G.M. (2005). Germ layer induction from embryonic stem cells. Exp. Hematol. *33*, 955–964.

Hemesath, T.J., and Stefansson, K. (1994). Expression of tenascin in thymus and thymic nonlymphoid cells. J. Immunol. *152*, 422–428.

Himburg, H.A., Muramoto, G.G., Daher, P., Meadows, S.K., Russell, J.L., Doan, P., Chi, J.T., Salter, A.B., Lento, W.E., Reya, T., et al. (2010). Pleiotrophin regulates the expansion and regeneration of hematopoietic stem cells. Nat. Med. *16*, 475–482.

Hsia, H.C., and Schwarzbauer, J.E. (2005). Meet the tenascins: multifunctional and mysterious. J. Biol. Chem. *280*, 26641–26644.

Hu, K., Yu, J., Suknuntha, K., Tian, S., Montgomery, K., Choi, K.D., Stewart, R., Thomson, J.A., and Slukvin, I.I. (2011). Efficient generation of transgene-free induced pluripotent stem cells from normal and neoplastic bone marrow and cord blood mononuclear cells. Blood *117*, e109–e119.

Jaffredo, T., Gautier, R., Brajeul, V., and Dieterlen-Lièvre, F. (2000). Tracing the progeny of the aortic hemangioblast in the avian embryo. Dev. Biol. *224*, 204–214.

Kazanskaya, O., Ohkawara, B., Heroult, M., Wu, W., Maltry, N., Augustin, H.G., and Niehrs, C. (2008). The Wnt signaling regulator R-spondin 3 promotes angioblast and vascular development. Development *135*, 3655–3664.

Keller, G. (2005). Embryonic stem cell differentiation: emergence of a new era in biology and medicine. Genes Dev. *19*, 1129–1155.

Kennedy, M., D'Souza, S.L., Lynch-Kattman, M., Schwantz, S., and Keller, G. (2007). Development of the hemangioblast defines the onset of hematopoiesis in human ES cell differentiation cultures. Blood *109*, 2679–2687.

Kennedy, M., Awong, G., Sturgeon, C.M., Ditadi, A., LaMotte-Mohs, R., Zúñiga-Pflücker, J.C., and Keller, G. (2012). T lymphocyte potential marks the emergence of definitive hematopoietic progenitors in human pluripotent stem cell differentiation cultures. Cell Rep *2*, 1722–1735.

Kissa, K., and Herbomel, P. (2010). Blood stem cells emerge from aortic endothelium by a novel type of cell transition. Nature *464*, 112–115.

Klein, G., Beck, S., and Müller, C.A. (1993). Tenascin is a cytoadhesive extracellular matrix component of the human hematopoietic microenvironment. J. Cell Biol. *123*, 1027–1035.

Marshall, C.J., Moore, R.L., Thorogood, P., Brickell, P.M., Kinnon, C., and Thrasher, A.J. (1999). Detailed characterization of the human aorta-gonad-mesonephros region reveals morphological polarity resembling a hematopoietic stromal layer. Dev. Dyn. *215*, 139–147.

Midwood, K.S., Hussenet, T., Langlois, B., and Orend, G. (2011). Advances in tenascin-C biology. Cell. Mol. Life Sci. *68*, 3175–3199.

Nakamura-Ishizu, A., Okuno, Y., Omatsu, Y., Okabe, K., Morimoto, J., Uede, T., Nagasawa, T., Suda, T., and Kubota, Y. (2012). Extracellular matrix protein tenascin-C is required in the bone marrow microenvironment primed for hematopoietic regeneration. Blood *119*, 5429–5437.

Ng, E.S., Davis, R.P., Azzola, L., Stanley, E.G., and Elefanty, A.G. (2005). Forced aggregation of defined numbers of human

embryonic stem cells into embryoid bodies fosters robust, reproducible hematopoietic differentiation. Blood *106*, 1601–1603.

Ng, E.S., Davis, R., Stanley, E.G., and Elefanty, A.G. (2008). A protocol describing the use of a recombinant protein-based, animal product-free medium (APEL) for human embryonic stem cell differentiation as spin embryoid bodies. Nat. Protoc. *3*, 768–776.

Nostro, M.C., Cheng, X., Keller, G.M., and Gadue, P. (2008). Wnt, activin, and BMP signaling regulate distinct stages in the developmental pathway from embryonic stem cells to blood. Cell Stem Cell *2*, 60–71.

Ohta, M., Sakai, T., Saga, Y., Aizawa, S., and Saito, M. (1998). Suppression of hematopoietic activity in tenascin-C-deficient mice. Blood *91*, 4074–4083.

Orend, G. (2005). Potential oncogenic action of tenascin-C in tumorigenesis. Int. J. Biochem. Cell Biol. *37*, 1066–1083.

Papadopoulos, N., Simopoulos, C., Sigalas, J., Kotini, A., Cheva, A., and Tamiolakis, D. (2004). Induction of hepatic hematopoiesis with tenascin-C expression during the second trimester of development. Eur. J. Obstet. Gynecol. Reprod. Biol. *113*, 56–60.

Pearson, S., Sroczynska, P., Lacaud, G., and Kouskoff, V. (2008). The stepwise specification of embryonic stem cells to hematopoietic fate is driven by sequential exposure to Bmp4, activin A, bFGF and VEGF. Development *135*, 1525–1535.

Pick, M., Azzola, L., Mossman, A., Stanley, E.G., and Elefanty, A.G. (2007). Differentiation of human embryonic stem cells in serum-free medium reveals distinct roles for bone morphogenetic protein 4, vascular endothelial growth factor, stem cell factor, and fibroblast growth factor 2 in hematopoiesis. Stem Cells *25*, 2206–2214.

Rafii, S., Kloss, C.C., Butler, J.M., Ginsberg, M., Gars, E., Lis, R., Zhan, Q., Josipovic, P., Ding, B.S., Xiang, J., et al. (2013). Human ESC-derived hemogenic endothelial cells undergo distinct waves of endothelial to hematopoietic transition. Blood *121*, 770–780.

Salvagiotto, G., Burton, S., Daigh, C.A., Rajesh, D., Slukvin, I.I., and Seay, N.J. (2011). A defined, feeder-free, serum-free system to generate in vitro hematopoietic progenitors and differentiated blood cells from hESCs and hiPSCs. PLoS ONE *6*, e17829.

Seiffert, M., Beck, S.C., Schermutzki, F., Müller, C.A., Erickson, H.P., and Klein, G. (1998). Mitogenic and adhesive effects of tenascin-C on human hematopoietic cells are mediated by various functional domains. Matrix Biol. *17*, 47–63.

Seki, M., Kameoka, J., Takahashi, S., Harigae, H., Yanai, N., Obinata, M., and Sasaki, T. (2006). Identification of tenascin-C as a key molecule determining stromal cell-dependent erythropoiesis. Exp. Hematol. *34*, 519–527.

Siewe, B.T., Kalis, S.L., Le, P.T., Witte, P.L., Choi, S., Conway, S.J., Druschitz, L., and Knight, K.L. (2011). In vitro requirement for periostin in B lymphopoiesis. Blood *117*, 3770–3779.

Slukvin, I.I. (2013). Deciphering the hierarchy of angiohematopoietic progenitors from human pluripotent stem cells. Cell Cycle *12*, 720–727.

Smith, B.W., Rozelle, S.S., Leung, A., Ubellacker, J., Parks, A., Nah, S.K., French, D., Gadue, P., Monti, S., Chui, D.H., et al. (2013). The aryl hydrocarbon receptor directs hematopoietic progenitor cell expansion and differentiation. Blood *122*, 376–385.

Soini, Y., Kamel, D., Apaja-Sarkkinen, M., Virtanen, I., and Lehto, V.P. (1993). Tenascin immunoreactivity in normal and pathological bone marrow. J. Clin. Pathol. *46*, 218–221.

Stewart, R., Rascón, C.A., Tian, S., Nie, J., Barry, C., Chu, L.F., Ardalani, H., Wagner, R.J., Probasco, M.D., Bolin, J.M., et al. (2013). Comparative RNA-seq analysis in the unsequenced axolotl: the oncogene burst highlights early gene expression in the blastema. PLoS Comput. Biol. *9*, e1002936.

Vodyanik, M.A., and Slukvin, I.I. (2007). Hematoendothelial differentiation of human embryonic stem cells. Curr. Protoc. Cell Biol. *Chapter 23*, 6.

Vodyanik, M.A., Bork, J.A., Thomson, J.A., and Slukvin, I.I. (2005). Human embryonic stem cell-derived CD34+ cells: efficient production in the coculture with OP9 stromal cells and analysis of lymphohematopoietic potential. Blood *105*, 617–626.

Vodyanik, M.A., Thomson, J.A., and Slukvin, I.I. (2006). Leukosialin (CD43) defines hematopoietic progenitors in human embryonic stem cell differentiation cultures. Blood *108*, 2095–2105.

Vodyanik, M.A., Yu, J., Zhang, X., Tian, S., Stewart, R., Thomson, J.A., and Slukvin, I.I. (2010). A mesoderm-derived precursor for mesenchymal stem and endothelial cells. Cell Stem Cell *7*, 718–729.

Wang, L., Li, L., Shojaei, F., Levac, K., Cerdan, C., Menendez, P., Martin, T., Rouleau, A., and Bhatia, M. (2004). Endothelial and hematopoietic cell fate of human embryonic stem cells originates from primitive endothelium with hemangioblastic properties. Immunity *21*, 31–41.

Wang, C., Tang, X., Sun, X., Miao, Z., Lv, Y., Yang, Y., Zhang, H., Zhang, P., Liu, Y., Du, L., et al. (2012). TGFβ inhibition enhances the generation of hematopoietic progenitors from human ES cell-derived hemogenic endothelial cells using a stepwise strategy. Cell Res. *22*, 194–207.

Zovein, A.C., Hofmann, J.J., Lynch, M., French, W.J., Turlo, K.A., Yang, Y., Becker, M.S., Zanetta, L., Dejana, E., Gasson, J.C., et al. (2008). Fate tracing reveals the endothelial origin of hematopoietic stem cells. Cell Stem Cell *3*, 625–636.

Stem Cell Reports, Volume *3*

Supplemental Information

Tenascin C Promotes Hematoendothelial Development and T Lymphoid Commitment from Human Pluripotent Stem Cells in Chemically Defined Conditions

Gene Uenishi, Derek Theisen, Jeong-Hee Lee, Akhilesh Kumar, Matt Raymond, Maxim Vodyanik, Scott Swanson, Ron Stewart, James Thomson, and Igor Slukvin

Supplementary Experimental Procedures

Flow Cytometry and FACS sorting

Flow Cytometry was conducted using the using a FACSCalibur flow cytometer (BD) and following antibodies: CD31-FITC (clone WM59), CD34-FITC (8G12), CD41a-FITC/APC (clone HIP8), CD43-FITC/PE/APC (clone 1G10), CD45-APC (clone HI30), CD73-FITC/PE (clone AD2), CD144-FITC/PE/AlexaFluor647 (clone 55-7H1), CD235a-FITC/PE/APC (clone GA-R2), KDR-PE/AlexaFluor647 (clone 89106), PDGFRα-PE (clone aR1) (BD Biosciences), TRA-1-85-FITC/PE (clone TRA-1-85), and APLNR-APC (clone 72133) (R&D Systems). Control staining with appropriate isotype-matched mouse monoclonal antibodies controls was included to establish threshold for positive staining and subset gating. Sorting was conducted on a FACS Aria (BD), as described previously (Choi et al., 2012; Vodyanik and Slukvin, 2007).

qPCR Assay

Cells were differentiated for 3 and 4 days and sorted on a FACSAria for the populations of interest from each day. RNA was collected using quick-RNA MiniPrep (Zymo Research) and quantified on a NanoDrop (GE Healthcare). Equal amounts of RNA was used for cDNA synthesis using SuperScript III First-Strand Synthesis System (Life Technologies). qPCR was conducted using Platinum SYBR Green qPCR SuperMix (Life Technologies) and primers sequences are listed below.

Gene	Accession No.	Primer Sequence	Amplicon length
ETV2	NM_014209	F: TCTTTGAAGCGGTACCAGAG	108
		R: GGGACCTCGGTGGTTAGTT	
FOXF1	NM_001451	F: AGCCGAGCTGCAAGGCATC	180
		R: CAGCCTCACATCACGCAAGG	
GATA2	NM_001145661	F: GGCGCACAACTAGATGGAA	100
		R: GGTTGGCATAGTAGGGGTTG	
MIXL1	NM_031944	F: AGTCCAGGATCCAGGTATGG	123
		R: TTTCAGTTCCAGGAGCACAG	
T	NM_003181	F: GACAATTGGTCCAGCCTTG	92

		R: GGGTACTGACTGGAGCTGGT	
RPL13a	NM_012423	F: CCACCCTGGAGGAGAAGAGG<br>R: CGTGGGTCTTGAGGACCTCTC	139

The reactions were run on a Mastercycler RealPlex Thermal Cycler (Eppendorf) and the expression levels were calculated by minimal cycle threshold values (Ct) normalized to the reference expression of RPL13a. The qPCR products were run on an agarose gel and stained with ethidium bromide to confirm specificity of the primers.

TCR Rearrangement Assay

Genomic DNA was isolated using quick-gDNA MiniPrep (Zymo Research). TCRβ and TCRγ clonality was detected using a PCR amplification kit (Invivoscribe) and AmpliTaq Gold DNA polymerase (Applied Biosystems) as previously described (Hu et al., 2011). The PCR products were analysed using heteroduplex analysis on a 6% polyacrylamide gel stained with ethidium bromide.

Microarray analysis of mouse stromal cell lines

A mouse bone marrow stromal cell line, OP9, was obtained from Dr. Toru Nakano (Research Institute for Microbial Diseases, Osaka University, Japan), S17 was obtained from Dr. Kenneth Dorshkind (University of California, Los Angeles) and MS-5 was obtained from the German Tissue Culture Collection. Stromal cell lines were cultured as previously described (Vodyanik et al., 2005). DNA-free RNA was isolated using RiboPure RNA kit and treated with DNAse using TURBO DNAfree reagents (Ambion). All samples were processed at the Gene Expression Center of the Biotechnology Center at the University of Wisconsin, Madison. To analyze the total stromal cell lines, we used standard arrays containing 60-mer probes manufactured

by NimbleGen Systems (Madison, WI), which contains ~385,000 60-mer probes, covering approximately 36,000 Mus musculus genome transcripts (HG17, NCBI Build 35). Microarrays were hybridized and processed following previously published protocols (Yu et al., 2007). Gene expression raw data were extracted using NimbleScan software v2.1. Considering that the signal distribution of the RNA sample is distinct from that of the gDNA sample, the signal intensities from RNA channels in all eight arrays were normalized with Robust Multiple-chip Analysis (RMA) algorithm (Irizarry et al., 2003). Separately, the same normalization procedure was performed on those from the mouse gDNA samples. For a given gene, the median-adjusted ratio between its normalized intensity from the RNA channel and that from the gDNA channel was then calculated as follows: Ratio = intensity from RNA channel/(intensity from gDNA channel + median intensity of all genes from the gDNA channel). To find which genes were uniquely upregulated in overgrown OP9 cells, first we first selected genes that showed at least a 3 fold higher expression in overgrown OP9 cells versus semi-confluent OP9 cells, overgrown OP9 cells versus MS5 stromal cells and overgrown OP9 cells versus S17 stromal cells. Then, we selected the genes that were common in all three comparison groups.

Supplemental References (Experimental Procedures):

Choi, K.D., Vodyanik, M.A., Togarrati, P.P., Suknuntha, K., Kumar, A., Samarjeet, F., Probasco, M.D., Tian, S., Stewart, R., Thomson, J.A., *et al.* (2012). Identification of the hemogenic endothelial progenitor and its direct precursor in human pluripotent stem cell differentiation cultures. Cell Rep *2*, 553-567.
Hu, K., Yu, J., Suknuntha, K., Tian, S., Montgomery, K., Choi, K.D., Stewart, R., Thomson, J.A., and Slukvin, II (2011). Efficient generation of transgene-free induced pluripotent stem cells from normal and neoplastic bone marrow and cord blood mononuclear cells. Blood *117*, e109-119.
Irizarry, R.A., Hobbs, B., Collin, F., Beazer-Barclay, Y.D., Antonellis, K.J., Scherf, U., and Speed, T.P. (2003). Exploration, normalization, and summaries of high density oligonucleotide array probe level data. Biostatistics *4*, 249-264.
Vodyanik, M.A., Bork, J.A., Thomson, J.A., and Slukvin, II (2005). Human embryonic stem cell-derived CD34+ cells: efficient production in the coculture with OP9 stromal cells and analysis of lymphohematopoietic potential. Blood *105*, 617-626.
Vodyanik, M.A., and Slukvin, II (2007). Hematoendothelial differentiation of human embryonic stem cells. Curr Protoc Cell Biol *Chapter 23*, Unit 23 26.
Yu, J., Vodyanik, M.A., Smuga-Otto, K., Antosiewicz-Bourget, J., Frane, J.L., Tian, S., Nie, J., Jonsdottir, G.A., Ruotti, V., Stewart, R., *et al.* (2007). Induced pluripotent stem cell lines derived from human somatic cells. Science *318*, 1917-1920.

Supplementary Figures

Supplementary Figure S1

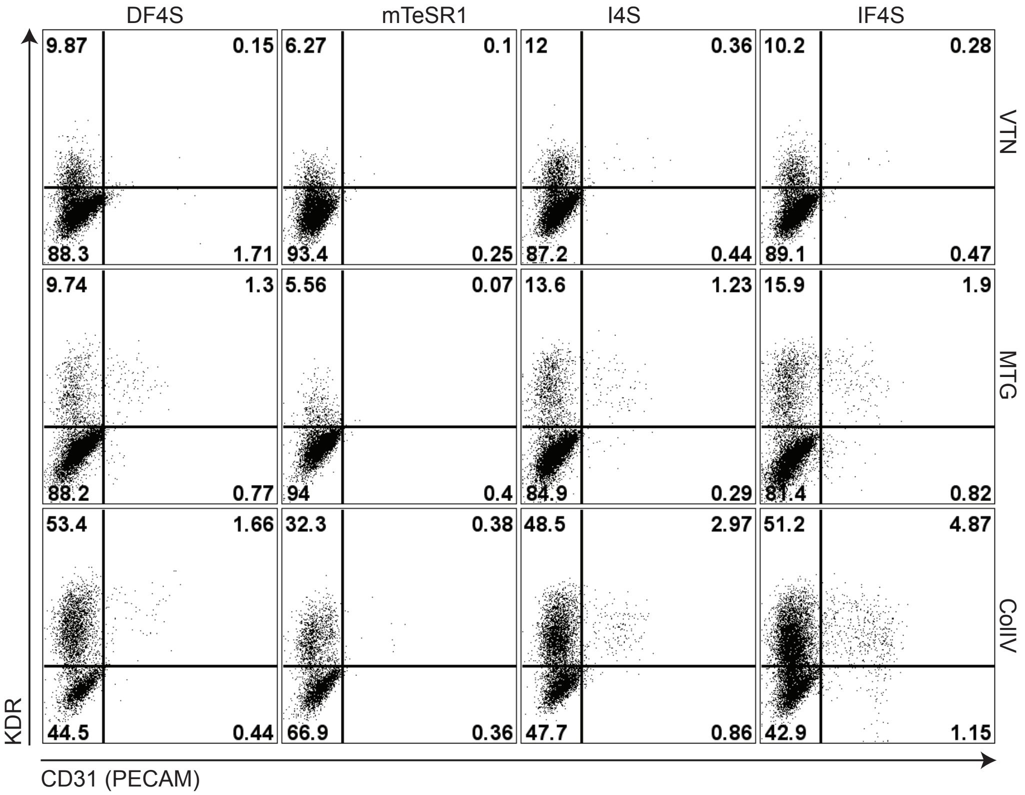

Supplementary Figure S1. Generation of $KDR^{+}CD31^{+}$ hematoendothelial progenitors in cultures using different basal media and matrix protein. mTeSR1 is mTeSR1 without FGF2 and TGFβ. DF4S is DMEM/F12-based media with 4 supplements: 64mg/L L-asorbic Acid 2-Phosphate $Mg2^{+}$ salt, 14μg/L sodium selenite, 10.6ng/L Holo-Transferrin, and 20mg/L Insulin. I4S is DF4S with IMDM-based media instead of DMEM/F12-based media, but with the 4 previously mentioned supplements. IF4S is DF4S with IMDM/F12-based media instead of DMEM/F12-based media, but with the 4 previously mentioned supplements. VTN is vitonectin matrix; MTG is Matrigel substrate; ColIV is Collagen IV matrix. Flow cytometry plots show percent of $CD31^{+}$ endothelial precursors of day 4 cells differentiated in each media supplemented with 50ng/ml FGF2, BMP4 and VEGF in hypoxia. Related to Figure 3.

Supplementary Figure S2

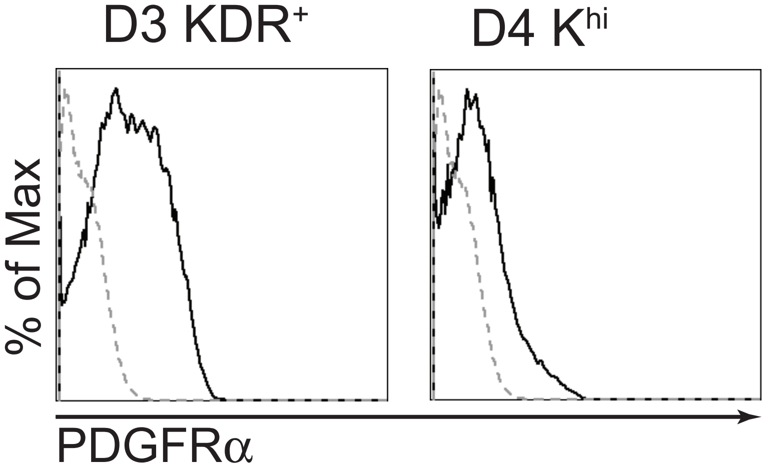

Supplementary Figure S2. Flow cytometry plot showing the expression of PDGFRα by gated day 3 KDR^{+} cells and by day 4 gated KDR^{high} (K^{hi}) cells. Related to Figure 3.

Supplementary Figure S3

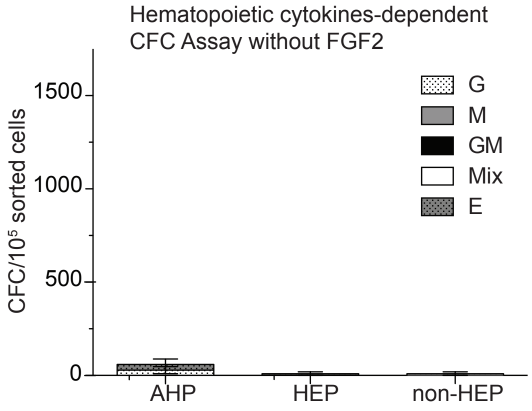

Supplementary Figure S3. CFC potential of isolated day 5 VEC^{+} subset in serum-free clonogenic medium containing hematopoietic cytokines without FGF2. AHP is VEC^{+}CD43/CD235^{+}CD73^{-} angiogenic hematopoietic progenitors, HEP is VEC^{+}CD43/CD235a^{-}CD73^{-} hemogenic endothelial progenitors, non-HEP VEC^{+}CD235a/CD43^{-}CD73^{+} non-hemogenic endothelial progenitors. Related to Figure 4.

Supplementary Figure S4

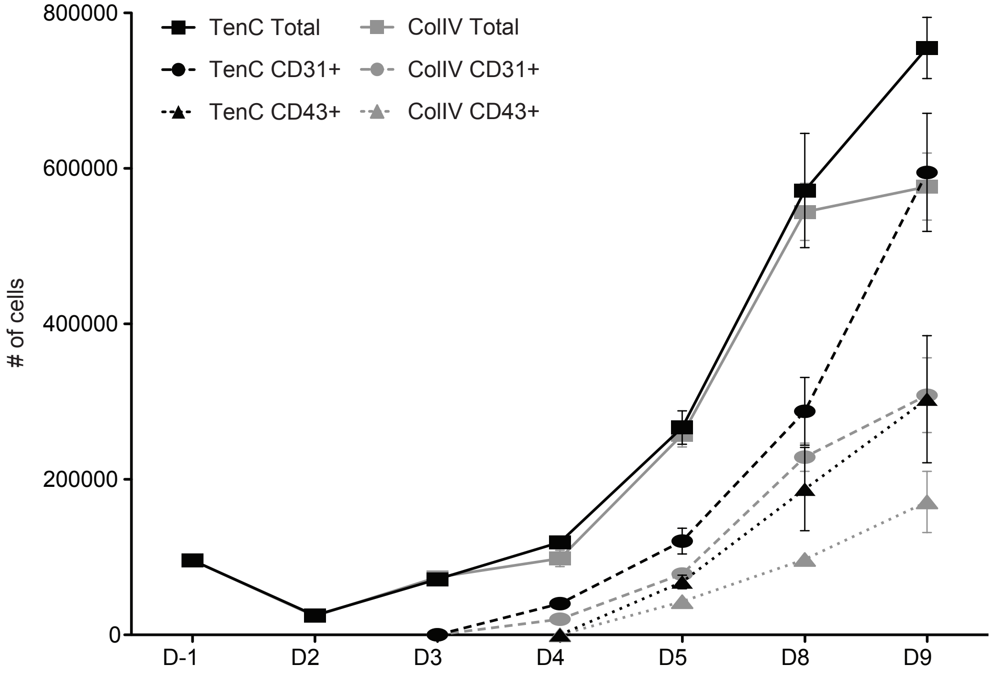

Supplementary Figure S4. Total number of cells generated from starting from day -1 when cells are plated on either TenC or ColIV, up to day 9 of differentiation. The numbers of CD31⁺ and CD43⁺ cells were calculated based on total number of cells times the percentage of positive cells based on flow cytometry. Results are mean ± SE of triplicates in one representative experiment. Related to Figure 4.

Supplementary Figure S5

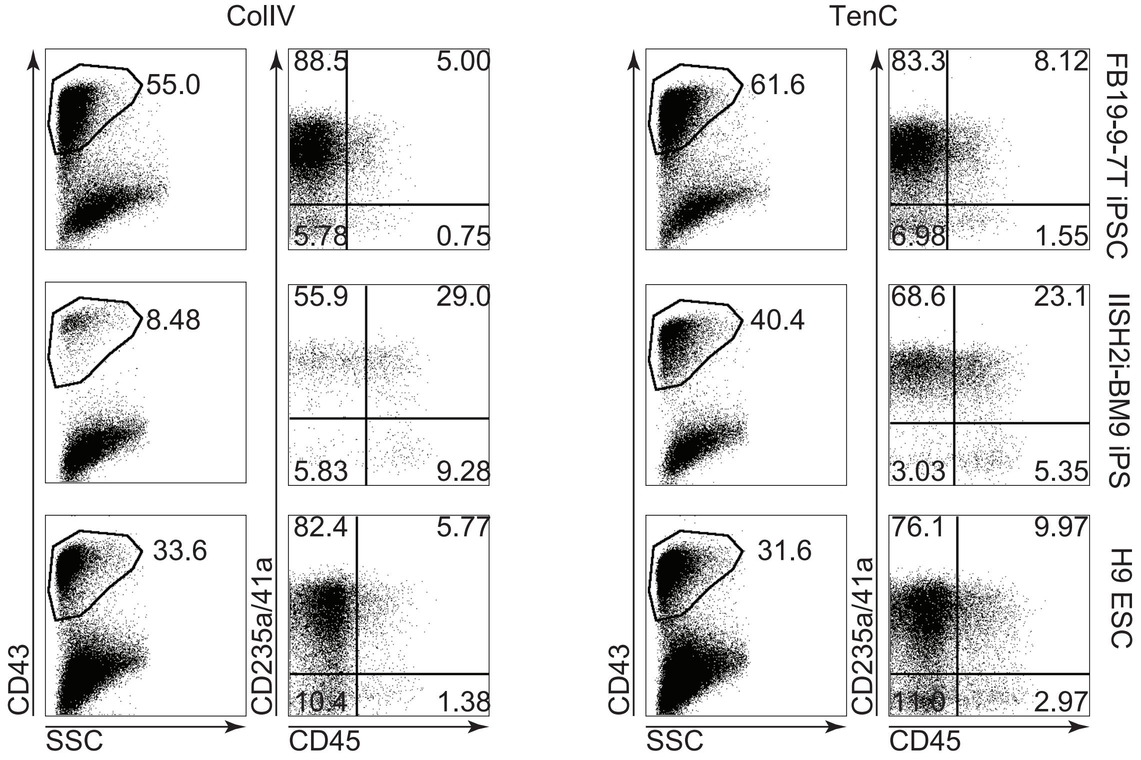

Supplemental Figure S5. Dot plots show the percentage of $CD43^+$ cells and their subsets of DF19-9-7T human fibroblast iPSC line, IISH2i-BM9 human bone marrow-derived iPSC line, and H9 human ESC line differentiated for 8 days on either ColIV or TenC. Related to Figure 5.

Supplementary Table S1. Complete Content of IF9S medium.

IF9S medium	Amount	Unit/L
**Inorganic Salts**		
Calcium Chloride	99.3	mg
Cupric Sulfate • 5H2O	0.00125	mg
Ferrous Sulfate • 7H2O	0.417	mg
Magnesium Chloride • 6H2O	28.8	mg
Magnesium Sulfate (anhydrous)	48.835	mg
Potassium Chloride	277	mg
Potassium Nitrate	0.038	mg
Sodium Bicarbonate	2100	mg
Sodium Chloride	6052	mg
Sodium Phosphate Dibasic (anhydrous)	71.02	mg
Sodium Phosphate Monobasic (anhydrous)	54.5	mg
sodium selenite	0.017	mg
Zinc Sulfate • 7H2O	0.4315	mg
**Amino Acids**		
glycin	26.255	mg
L-alanyl-L-glutamine dipeptide	434.4	mg
L-alanine	25.9	mg
L-Arginine • HCl	160.7	mg
L-asparagine	21.705	mg
L-Aspartic acid	34.95	mg
L-Cysteine • HCl • H2O	17.5	mg
L-Cystine • 2HCl	45.62	mg
L-Glutamic Acid	59.55	mg
L-Glutamine	365	mg
L-Histidine • HCl • H2O	31.48	mg
L-Isoleucine	54.47	mg
L-Leucine	59.05	mg
L-Lysine • HCl	91.25	mg
L-Methionine	17.24	mg
L-Phenylalanine	35.48	mg
L-proline	48.75	mg
L-Serine	36.75	mg
L-Threonine	53.45	mg
L-Tryptophan	9.02	mg
L-Tyrosine • 2Na • 2H2O	55.785	mg
L-Valine	52.85	mg
**Vitamins**		
D-Biotin	0.01015	mg
Choline Chloride	8.98	mg
Folic Acid	2.66	mg
holo-transferrin	10.7	mg
myo-Inositol	12.6	mg
Niacinamide	2.0185	mg

D-Pantothenic Acid (hemicalcium)	2.24	mg
Pyridoxal • HCl	2	mg
Pyridoxine • HCl	0.031	mg
Riboflavin	0.219	mg
thiamine	2.17	mg
Vitamin B12	0.6865	mg
**Other**		
D-Glucose	3151	mg
HEPES	2979	mg
Hypoxanthine	2.04	mg
Phenol Red • Na	8.65	mg
Putrescine • HCl	0.0805	mg
DL-Thioctic Acid	0.105	mg
Pyruvic Acid • Na	110	mg
Thymidine	0.365	mg
1-thioglycerol	40	ul
polyvinyl alcohol	10	g
insulin	20	mg
L-ascorbic acid 2-phosphate magnesium salt	64	mg
**Lipids**		
arachidonic acid	0.004	mg
cholesterol	0.44	mg
DL-alpha-Tocopherol Acetate	0.14	mg
Linoleic Acid	0.0956	mg
Linolenic Acid	0.02	mg
Myristic Acid	0.02	mg
Oleic Acid	0.02	mg
Palmitic Acid	0.02	mg
Palmitoleic Acid	0.02	mg
Pluronic F-68	180	mg
Stearic Acid	0.02	mg
Tween-80	4.4	mg
